# Characterization of 2D precision and accuracy for combined visual-haptic localization

**DOI:** 10.3389/fnins.2025.1528601

**Published:** 2025-03-12

**Authors:** Madeline Fischer, Umberto Saetti, Martine Godfroy-Cooper, Douglas Fischer

**Affiliations:** ^1^Department of Mathematics, University of Maryland, College Park, MD, United States; ^2^Department of Aerospace Engineering, University of Maryland, College Park, MD, United States; ^3^AIRBUS, Toulouse, France; ^4^The Boeing Company, Ridley Park, PA, United States

**Keywords:** multisensory integration, haptic cueing, visual cueing, multi-modal cueing, situational awareness, localization, aviation, degraded visual environment

## Abstract

This article describes a combined visual and haptic localization experiment that addresses the area of multimodal cueing. The aim of the present investigation was to characterize two-dimensional (2D) localization precision and accuracy of visual, haptic, and combined visual-tactile targets in the peri-personal space, the space around the body in which sensory information is perceived as ecologically relevant. Participants were presented with visual, haptic, or bimodal cues using the body-centered reference frame and were instructed to indicate the corresponding perceived target location in space using a mouse pointer in an open-loop feedback condition. Outcomes of the unimodal (visual and haptic) and bimodal (combined visual-haptic) localization performance were used to assess the nature of the multisensory combination, using a Bayesian integration model. Results of the study revealed that the visual and haptic perceptive fields are characterized differently in terms of localization performance, providing important considerations for the transformation of each sensory modality when combining cues into a unified percept. The results reaffirmed many well known radial characteristics of vision with respect to localization, and identified a nonlinear pattern of haptic localization performance that was largely influenced by the midline of the center of the torso and each side of the cutaneous region. Overall, the lack of improvement in precision for bimodal cueing relative to the best unimodal cueing modality, vision, is in favor of sensory combination rather than optimal integration predicted by the Maximum Likelihood Estimation (MLE) model. Conversely, the hypothesis that accuracy in localizing the bimodal visual-haptic targets would represent a compromise between visual and haptic performance in favor of the most precise modality was rejected. Instead, the bimodal accuracy was found to be equivalent to or to exceed that of the best unimodal condition, vision. The results provide some insight into the structure of the underlying sensorimotor processes employed by the brain and confirm the usefulness of capitalizing on naturally occurring differences between vision and haptic to better understand their interaction and their contribution to multimodal perception These results will help inform the development of future human-machine interfaces implementing haptic feedback mechanisms In the context of pilot performance, haptic localization can have several benefits including enhanced situational awareness, improved spatial orientation, reduced workload, thereby contributing to safer operations. These benefits can be applied to future systems for aircraft handling by helping overcome visual illusions and discrepancies between visual and vestibular sensory channels, especially in degraded visual environments.

## 1 Introduction

Approximately 80% of all aircraft mishaps are attributed to human error, as reported by the Federal Aviation Administration (Anon, 2023). Human error encompasses all inappropriate human behaviors that lower system effectiveness or safety, which may be triggered or influenced by a number of environmental factors, system characteristics, and human abilities (Lee et al., 2017). Human factors engineers aim to improve human interactions with systems, with particular emphasis on complex systems that operate in highly dynamic environments, such as aviation (Lee et al., 2017; Fischer et al., 2023). Vertical lift vehicles are characterized by unstable, high-order, and highly-coupled dynamics across most of their restricted flight envelopes due to complex power and structural limits. Moreover, these vehicles often operate in high-risk and degraded visual environments (DVEs). All of these factors contribute significantly to increase pilot cognitive and physical workload, and interfere with the pilots' ability to detect and interpret information in the external environment. Perceptual errors involving sensory information that is either incorrectly perceived or not perceived by the users will hinder their ability to efficiently process the information, decide the necessary actions, and ultimately act on a given scenario. While operating in DVEs, pilots are particularly susceptible to illusions such as spatial disorientation, which involve a discrepancy in the perceived angular motion between visual and vestibular systems (Lee et al., 2017). To overcome such illusions and minimize corresponding pilot errors, systems may be adapted using strategies such as multimodal cueing to provide complementary or redundant signals to the user that help overcome deficiencies created when information is denied in a particular sensory modality, or is discordant across perceptual modalities.

*Multisensory integration* is the transformation of unisensory inputs into a multisensory product that is unique from its component (*i.e*., unisensory) parts. This applies to both the physiological process that takes place at the level of individual multisensory neurons and the behavioral consequences of this neural process (Stein and Rowland, 2020; van Erp, 2005, 2007; Godfroy-Cooper et al., 2015). To enhance the estimate of an environmental property like the position of an object in space (Where) or the nature of this object (What), redundant or complementary signals may be combined into a single *unified percept*, and processed as one event rather than separate events, assuming cognitive, spatial and temporal congruency (Tong et al., 2020; Driver and Spence, 1998; Spence, 2013; Goktepe et al., 2024). In this way, redundant information creates a more robust percept, in that if sensory information from one modality is not available (*i.e*., in DVEs), then information from another source may substitute (Ernst, 2006; van Erp, 2005; Welch, 1999; Roach et al., 2006). This leads to phenomena described as for example the “Principle of Inverse Effectiveness” in multisensory integration which states that, as the responsiveness to individual sensory stimuli decreases, the strength of multisensory integration increases (Holmes, 2009).

The fundamental concept of multisensory integration is that the bandwidth of human-machine communication can be increased by optimizing information processing and ensuring that sensory information in each modality fits intuitively within the context of the task demand and has semantic consistency across modalities (Hancock et al., 2015; van Erp, 2005). It encompasses the interactions, conflicts, and biases caused by the processing of information in different modalities, including the combination of sensory inputs into a unified percept (Godfroy-Cooper et al., 2015).

While spatial visual-auditory integration has been studied at large (Godfroy-Cooper et al., 2015; Odeggard et al., 2015; Opoku-Baah et al., 2021; Frens et al., 1995; Colonius and Dietrich, 2010), there remains a need for investigating the possibilities of incorporating tactile cueing mechanisms into the existing multisensory integration models.

*Tactile* or *haptic cueing* is defined as the process of delivering information through the sense of touch (Hancock et al., 2015). Results from previous studies suggest that the human sense of touch involves a tactile field, analogous to the visual field. The tactile field supports computation of spatial relations between individual stimulus locations, and thus underlies tactile pattern perception (Haggard and Giovagnoli, 2011).

Tactile stimuli may be delivered in the form of vibration, pressure, temperature, kinesthetics, and electrical stimulation, among others. The amount and process by which stimuli are noticed, or *tactile salience*, determines the relative prominence of specific stimuli and varies depending on the characteristics of the stimuli as well as the task (Hancock et al., 2015). Within the context of aviation, the use of haptic feedback has shown benefits for the formation of motor-memory and support for certain temporal tasks (Deldycke et al., 2018; Fabbroni, 2017; D'Intino et al., 2018; Olivari et al., 2014b; D'Intino et al., 2020a; Olivari et al., 2014a; Fabbroni et al., 2017b; D'Intino et al., 2020b; Fabbroni et al., 2017a). Moreover, the combination of haptic and spatial audio cues were shown to enhance situational awareness (Reynal et al., 2019; de Stigter et al., 2007; Brill et al., 2014; Tzafestas et al., 2008; Miller et al., 2019; Godfroy-Cooper et al., 2018; Begault et al., 2010; Wenzel and Godfroy-Cooper, 2021), localization (Godfroy-Cooper et al., 2015; Deneve and Pouget, 2004; Angelaki et al., 2009), flight envelope protection (Van Baelen et al., 2020; Müllhäuser and Leiling, 2019; Müllhäuser and Lusardi, 2022; Sahasrabudhe et al., 2006; Jeram and Prasad, 2005), and pilot-vehicle system performance performance (McGrath, 2000; McGrath et al., 2004; Wolf and Kuber, 2018; Jennings et al., 2004; Morcos et al., 2023a,b, 2024).

None of the studies above, however, investigate the use of haptics for localization tasks. Localization, or the ability to determine a position in space, can be used to illustrate the encoding mechanism of multi-sensory integration by utilizing the body-centered reference frame to detect a signal in the peri-personal space (PPS) (Heed et al., 2015; Matsuda et al., 2021; Brill et al., 2019; Rossi Sebastiano et al., 2022). Presenting information as a combination of multiple sensory sources requires an intuitive somatotopic mapping of direction and spatial orientation information (van Erp, 2007). In a previous study (Godfroy-Cooper et al., 2015), conducted a 2D localization experiment investigating visual, auditory, and combined bimodal cueing mechanisms with respect to 2D precision and accuracy. In this study, participants were presented with target cues from each modality that were spatially and temporally congruent, and instructed to indicate the corresponding perceived location in space using a mouse pointer on a visual response plane. Similar to what was done in Godfroy-Cooper et al. (2015) with auditory targets, the current study incorporated tactile cueing in combination with visual cueing in order to characterize the unimodal perceptual space related to haptic cueing and further investigated the multisensory integration phenomenon.

The objectives of the study were to: (i) characterize visual and haptic localization in terms of precision and accuracy with respect to a body-centered reference frame, and (ii) use the respective visual and haptic localization precision and accuracy to test a model of optimal integration. To achieve these objectives, an experiment was designed where participants were instructed to report visually, the perceived position of a visual, haptic or bimodal visual-haptic stimulus displayed at a fixed distance in two-dimensional (2D) space. This human psychophysics experiment was performed in which an egocentric localization paradigm was chosen to test a Bayesian model of cue integration of spatially and temporally congruent visual and haptic stimuli.

The article begins with a mathematical description of the statistical theory underlying the multisensory integration principles (Bresciani et al., 2006; Ghahramani, 1995; Shams et al., 2005; Roach et al., 2006; Koerding and Wolpert, 2006; Koerding et al., 2007). This section is followed by an explanation of the participants, apparatus, targets and procedure. An in-depth description of the variables used to describe precision and accuracy as measures of performance is provided as well. The following statistical analysis outlines the methods for characterizing each unimodal space, testing the maximum likelihood estimation (MLE) model, and determining performance enhancement patterns of the bimodal condition. Results of the analyses (i) validate and expand prior knowledge about visual and haptic perceptive fields, (ii) test the traditional model of cue integration (Ghorbani, 2019) assuming a maximum-likelihood estimation (MLE), and (iii) identify important patterns for the cognitive processing methods used by cues from each sensory modality. Results of the MLE predictions are compared for each condition, with observed significant areas of enhanced performance described that were predicted but not observed. Relationships between precision and accuracy, as well as metrics of redundancy gain provide information for methods that may effectively improve the ability to predict when multimodal cueing is most effective. Following the results, a discussion connects findings of the present study to the literature, and identifies possible limitations. The article is concluded with a summary of the study's overall findings.

## 2 Materials and methods

### 2.1 Mathematical background

The theory of multisensory integration aims to optimally combine sensory percepts as a weighted average of information from each source. According to signal detection theory, there is some variance associated with any estimate of an environmental property that corresponds to the reliability of the source (Ernst, 2006). Each estimate is assumed to be independent of one another and approximately normally distributed around the true mean such that if Ŝ is the estimated environmental property, then $\hat{S}\sim N\left(\bar{S},\sigma^{2}\right)$. The variance associated with each estimate, σ^2^ corresponds to a measure of reliability for the estimate such that *r* = 1/σ^2^.

The MLE theory of multisensory integration mathematically explains the relationship between the reliability of a source and its effect on the sensory interpretation of another source (Van Dam and Ernst, 2014). In general, MLE methods build an estimator for a parameter of interest by solving for the maximum value of the likelihood function for that parameter (Carlin, 2009; Casella and Berger, 2002). Here, we are interested in estimating the 2D localization of visual-haptic stimuli using the reliability of each unimodal condition involved. We capitalized on the inherent variations of spatial resolution of visual and haptic modalities with eccentricity and direction in the frontal field to “manipulate” the relative reliability of each sensory modality.

We denote visual (V), haptic (H), and visual-haptic (VH) as the unimodal and multimodal conditions of interest. Hence, the MLE theory defines the multimodal localization estimate, Ŝ_VH_, as a weighted average of each unimodal estimate according to their location estimate and the weight developed from their precision measurement such that:


(1) $$\begin{array}{l} \hat{S}=\underset{i}{\sum }W_{i}{\hat{S}}_{i} \end{array}$$

where Ŝ is the overall estimate, *i* denotes the unimodal V or H condition, *W*_*i*_ is the weight of each unimodal condition, and the sum of unimodal weights must satisfy $\underset{i}{\sum }W_{i}=1$. The weights assigned to each sensory source are proportional to the reliability of the signal, such that the more reliable the signal is, the heavier the weight. In this manner, it predicts the joint reliability estimate of the multi-modal condition as a combination of each sensory modality and their respective reliability estimate. Given that the reliability of a particular source could influence the reliability and interpretation of another source, the MLE model predicts that the more precise of two modalities will bias the less precise (Welch and Warren, 1980; Godfroy-Cooper et al., 2015).

Applying the Bayesian method to multisensory integration theory allows to determine each relative weight as a function of prior variances. In general, Bayesian methods combine prior knowledge with some given data and processes it in order to make an inference about a certain population (Carlin, 2009). The Bayesian model for multisensory integration aims to predict in which condition the sensory estimate of a multimodal percept will exceed that of the most precise unimodal condition (Godfroy-Cooper et al., 2015; Colonius and Diederich, 2020). For the multimodal localization task, the Bayesian method predicts that combined visual-haptic localization will exceed that of the more precise modality (typically vision) (Godfroy-Cooper et al., 2015) according to:


(2) $$\begin{array}{l} \sigma_{\text{VH}}^{2}=\frac{\sigma_{\text{V}}^{2}\sigma_{\text{H}}^{2}}{\sigma_{\text{V}}^{2}+\sigma_{\text{H}}^{2}} \end{array}$$

where $\sigma_{\text{VH}}^{2},\sigma_{\text{V}}^{2},\sigma_{\text{H}}^{2}$ are variances in the bimodal or unimodal conditions, respectively. From the variances of each condition, the weight of sensory information from each unimodal condition is derived as the normalized reciprocal variances of the unimodal conditions as follows:


(3a-b) $$W_{\text{V}}\frac{\frac{1}{\sigma_{\text{V}}^{2}}}{\frac{1}{\sigma_{\text{V}}^{2}}+\frac{1}{\sigma_{\text{H}}^{2}}},\;W_{\text{H}}\frac{\frac{1}{\sigma_{\text{H}}^{2}}}{\frac{1}{\sigma_{\text{V}}^{2}}+\frac{1}{\sigma_{\text{H}}^{2}}}$$

The respective weights are applied to the location estimate for each target in each modality in order to produce a predicted measure of accuracy as:


(4) $$\begin{array}{l} {\hat{r}}_{\text{VH}}=r_{\text{V}}W_{\text{V}}+r_{\text{H}}W_{\text{H}} \end{array}$$

where *r*_V_ and *r*_H_ are the observed visual and haptic location estimates.

### 2.2 Methodology

#### 2.2.1 Participants

Sixteen healthy adults, fourteen men and two women, were enrolled in the experiment based on a power analysis according to a repeated measures, within subject design with moderate effect size, assumed correlation coefficient 0.5, 35 measures (targets), and a required power of 0.8 for minimum statistical significance. Participants were all volunteers aged 22 to 33 years old (μ : 26.19, σ : 3.53) from the University of Maryland and Washington D.C. region. All participants had normal visual, auditory and haptic sensitivity allowing for normal age-related differences according to the following criteria: 20/20 visual acuity (corrected if necessary), and no known hearing impairments or nerve damage. Additionally, all participants were free from any known or diagnosed history of neurological or muscular skeletal disabilities. Waist circumference (ranged 72 − 115.5 cm, μ = 86.69, σ = 11.98), hand dominance (14 R, 2 L) and eye dominance (15 R, 1 L) were recorded to make note of possible body differences.

This experiment was carried out in accordance with the Institutional Review Board (IRB) and all measures were taken to protect the confidentiality and safety of participants. All subjects provided written consent in accordance with IRB protocol prior to participating in the experiment.

#### 2.2.2 Apparatus

The experimental apparatus used was designed to replicate that used in an earlier study (Godfroy-Cooper et al., 2015) which investigated the effect of spatial determinants on visual-auditory integration. The research compared the effect of direction and eccentricity on the localization of spatially and temporally congruent visual-auditory stimuli, capitalized on the inherent variations in localization precision and accuracy as a function of spatial location.

The participant sat on a still, non-rotating stool in front of a semi-cylindrical screen and placed their head on a chin rest with forehead resting against an attached headband to keep the head still. The screen used to display target points and accept visual responses was a Samsung Odyssey Ark, which has a 55 inch curved screen monitor with 1000R curvature (*i.e*., where the monitor would form a perfect circle with a radius of 1, 000 mm). The distance between the screen and the participant's eyes was 1 meter in the horizontal direction to ensure equal distance from each of the target points in azimuth. Note that the screen was not curved in the vertical direction.

**Figure 1:**
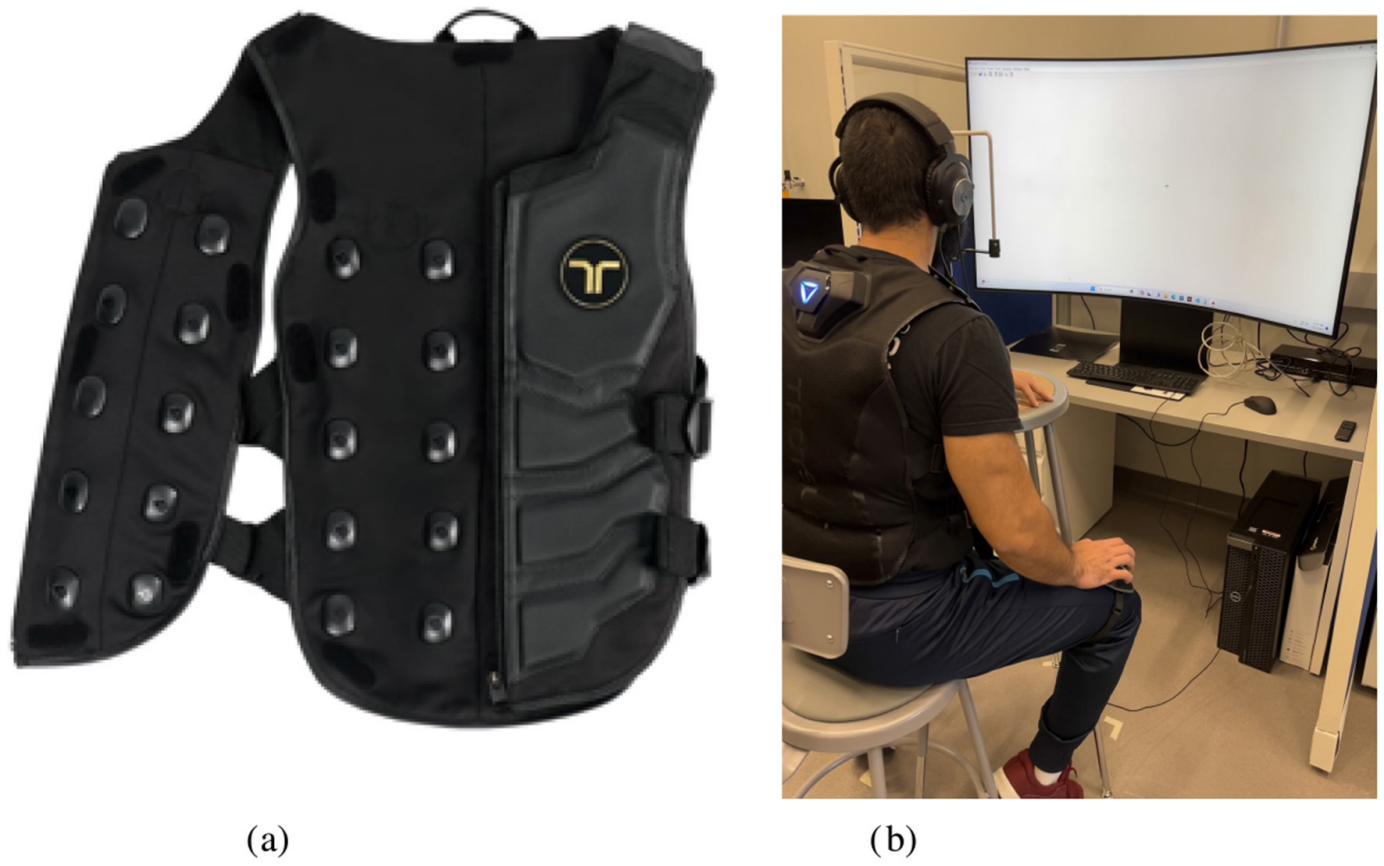
Experimental setup and equipment. **(A)** Tactsuit X40. **(B)** Experimental apparatus.

Haptic stimuli were delivered using the bHaptics Tactsuit X40, as shown in Figure 1. This haptic vest uses 20 eccentric rotating mass (ERM) motors on the front torso region to deliver pressure cues perceived as vibrations. The adjustable parameters of the suit are power – the intensity of the signal, timing—the constant time (in milliseconds, or ms) or speed between each point in the vibration pattern, length – the time (in ms) of the entire feedback pattern, and feedback intensity—a percentage representing the adjustable intensity of the perceived signal. Frequency of the signal ranges from 20 to 120 Hz depending on the intensity of the vibration. Haptic tactors were not visible to the participant, and no feedback was delivered following their response to a presented stimuli.

#### 2.2.3 The targets

**Figure 2:**
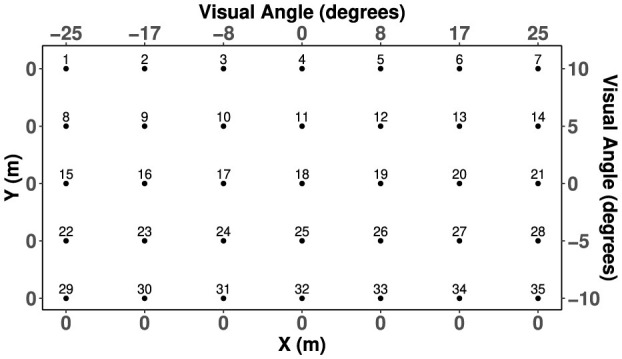
Target positions in the cartesian coordinate plane. Target 18 represents the “origin” target, which was aligned with each participant's direct center of gaze.

Four levels of eccentricity in azimuth and three levels in elevation were assessed using 35 target points, which had symmetry around the horizontal median plane (HMP, 0° in elevation) and sagittal median plane (SMP, 0° in azimuth). These 35 target points organized visually in a 5 × 7 grid plane according to the map in Figure 2. In each modality, corresponding targets were constructed to represent the same hypothetical location as the target on the visual field. Each target point was attached to a target identity number corresponding to a grid point on the visual plane and a haptic (vibration) cue produced by specific combinations of tactors. When combined, the visual and haptic targets achieved spatial and temporal congruency.

In the present experiment, spatial congruency refers to perceptually overlapping stimuli. The stimuli are not physically spatially congruent *per se*, as the V stimulus is presented at a distance from the body, and encoded in a retinotopic frame of reference, while the H stimulus is perceived at the surface of the body and encoded in a trunk-centric reference frame. The inherent difficulty with using haptics to depict a direction and hence a location in space is that this requires a transformation, here a projection, assuming a trunk-centered reference frame.

A fixation cross was used to orient the participant to “zero,” or the center point, on the visual plane. This cross corresponds to a similar “zero” cue in the haptic modality located at the center point of the suit vest, which was simultaneously presented with the visual cross to orient and indicate to the participant where the center of the reference frame is located in each modality.

With respect to the multisensory integration hypothesis, presenting spatially congruent cues in multiple sensory modalities should increase the localization ability of humans. Cues in visual and haptic modalities are encoded and localized differently according to individual differences in physical anthropometric measures and cognitive processing abilities. In order to achieve this spatial congruence, cues were transformed according to the manner in which the source is localized in each modality.

**Figure 3:**
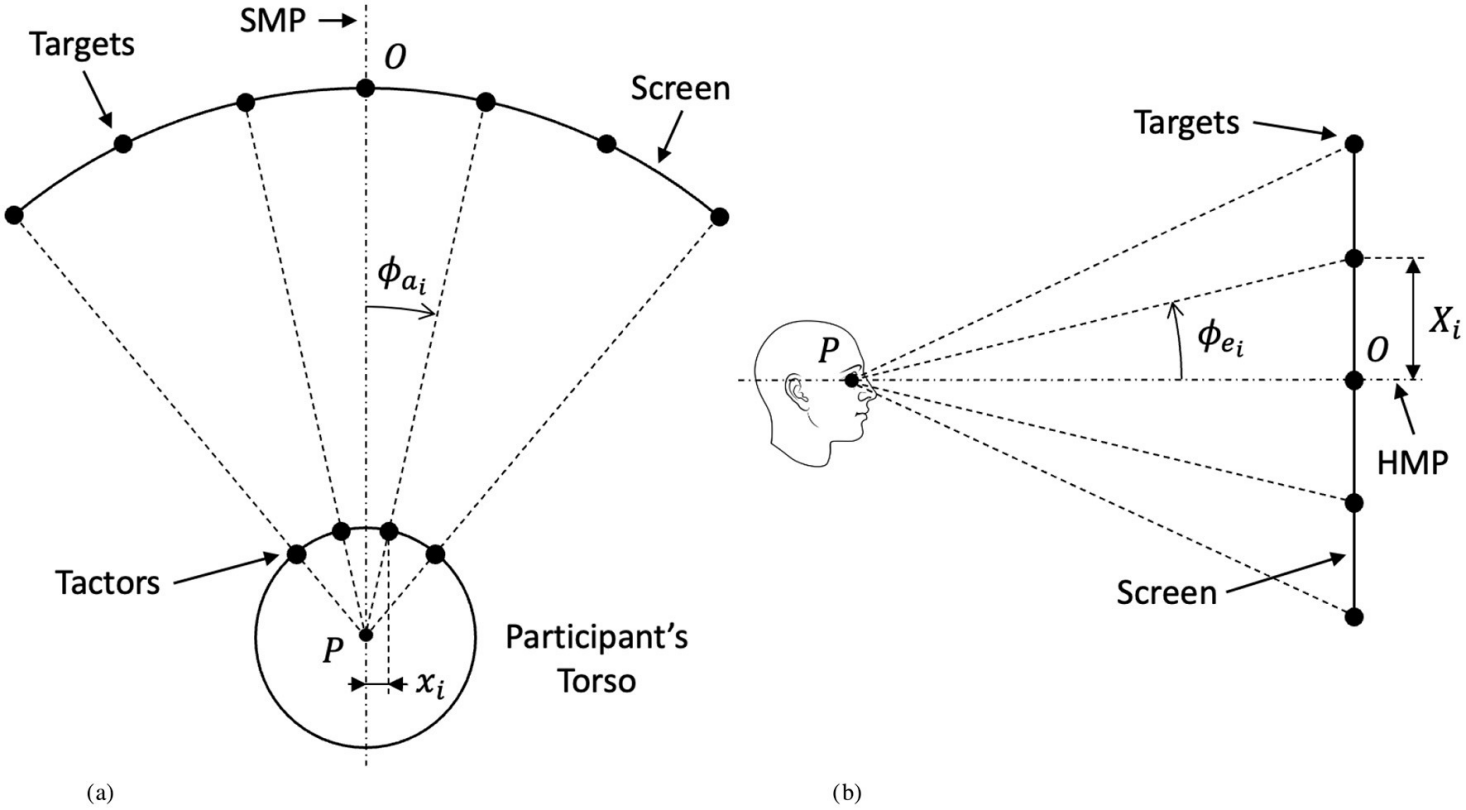
Experiment geometry explaining the correspondence between the position of haptic targets and their corresponding visual counterparts on the testing screen. **(A)** Top view (coincident with the horizontal median plane). **(B)** Side view (coincident with the sagittal median plane).

First, the haptic target cues were produced. The Tactsuit X40 contains a 4 × 5 grid of tactors on the front torso region. The 35 haptic target cues used in the experiment were developed using a gradient function that drew intensity from surrounding tactors to create 35 simulated discrete target points. The position of the visual cues were determined as a projection from the location of the target on the suit to the curved screen in front of the participant. According to Asseman et al. (2008), expected direction of gaze in azimuth in response to a tactile stimulus on the front torso can be determined as a ratio of distance of the stimulus from the mid-line and total waist circumference. In this manner, the expected visual gaze angle, ϕ_*a*_, is used to determine locations for corresponding visual cues and is calculated under the assumption that waist circumference is equivalent to a perfect cylinder as it is mapped to the visual plane. Waist measurements for the 50^th^ percentile male and female, according to the U.S. Army anthropometric measures (Gordon et al., 2016), were used to produce visual gaze angles for each target point for both male and females. The visual gaze angle is defined (in radians) as (Asseman et al., 2008):


(5) $$\begin{array}{l} \phi_{a_{i}}\approx 2\pi \frac{x_{i}}{C} \end{array}$$

where *x*_*i*_ is the distance of the *i*^th^ tactor from the midline of the torso and *C* is the total waist circumference. The visual gaze angle is shown qualitatively in Figure 3A.

Angles in elevation were calculated as a projection from haptic cues in order to maintain equal spacing. Since target points on the haptic suit are fixed and equally spaced in both azimuth and elevation, the corresponding visual targets were equally spaced in elevation as well. The angles in azimuth calculated above were used to determine the distance between target points on the screen:


(6) $$\begin{array}{l} X_{i}=r_{P\to O}\phi_{a_{i}} \end{array}$$

where *X*_*i*_ is the distance between visual targets and the mid-line on the screen and *r*_*P*→*O*_ is the distance of the participant (point *P* in Figure 3) from the screen (point *O* in Figure 3)—in our case, 1 meter. From the resulting *X*_*i*_ distances of targets on the screen, the visual angles in elevation, θ were determined according to:


(7) $$\begin{array}{l} \phi_{e_{i}}=\tan^{-1}\left(\frac{X_{i}}{r}\right) \end{array}$$

to produce the resulting range of angles in elevation. The visual elevation angle is shown qualitatively in Figure 3B. Target positions were then scaled to fit the size of the monitor used in the experiment, resulting in visual angle positions of approximately ϕ_*a*_ = {±25, ±17, ±8, 0} degrees in azimuth and ϕ_*e*_ = {±10, ±5, 0} degrees in elevation for both males and females. The exact layout of target points for both males and females labeled by target identification (ID) and with their respective visual spacing and angles of separation are displayed in Figure 2.

The visual (V) target was a black circular dot (1 deg visual angle that appeared for 100 ms. The contrast between the visual target dot and the white background was designed to optimize signal detection across the visual field. The haptic target cue was a vibration delivered by a combination of vibro-tactile motors on the Tactsuit for a duration of 100 ms. Twenty vibro-tactile motors are used on the front torso region of the Tactsuit, which are arranged in a 5 × 4 grid. In order to create 35 target cues that correspond to the visual target field, a gradient function was used to draw power of varying intensities from surrounding motors. The relative intensity of the cue in each modality was designed to fall around a 6 on a subjective Likert scale from 1-10 so that each signal was highly discriminable but never painful or uncomfortable. This relative intensity rating of each signal was assessed through a subjective measure of perceived intensity test, described in the procedure below.

#### 2.2.4 Procedure

In the localization experiment, participants were presented with a visual, haptic, or visual-haptic target and subsequently directed to indicate the perceived target location in space using a mouse pointer in an open loop feedback condition. This way, they indicated a target location on a 2D visual coordinate plane that corresponded to a signal that was either seen on the screen felt on their body, or seen and felt together. Target cues were presented in a total of 3 conditions spanning all unimodal and bimodal combinations: visual (V), haptic (H), and visual-haptic (VH).

The first phase of the experiment assessed the perceived intensity of the signals delivered to participants in each modality. Participants were presented with isolated signals in either the visual or haptic modality. In the same manner as the testing session, the target cue was presented at the origin location of each modality for a duration of 100 ms. After the offset of the signal, participants were asked to rate the intensity of the signal according to given a reference frame with 1 corresponding to “just perceived signal” and 10 corresponding to “would not want to feel the signal again.” The scale was developed based on similar experiments (Diamant and Reilly, 2011; Reilly, 1998) that investigated the tolerance of perceived pressure or electrically induced haptic stimuli. In each modality, the intensity of the signal was designed to fall around a 6 out of 10 to be highly discriminable but never painful or uncomfortable. The results from this phase were used solely to ensure that the signals were clear, unobstructed, and easily perceivable by all participants. No further analysis was conducted on the perceived intensities, as this phase was intended only as a preliminary step to validate the stimuli for the subsequent phases of the experiment.

During the second phase of the experiment, the learning phase, participants learned the mapping of each haptic cue to the visual response plane with respect to the body-centered reference frame. During this phase, participants received haptic signals simultaneously with the appearance of the corresponding target in the visual plane. Participants were instructed to use the mouse pointer to select the target location on the screen immediately after they perceived the target signal. Participants completed this calibration procedure for a randomized order of each of the boundary and midline points of the target grid in order to learn the edges of the target space in each modality.

The final phase of the experiment, the testing phase, assessed 2D localization performance with respect to precision and accuracy in each modal condition. A fixation cross was presented at the center of the screen for a random period of 500–1, 500 ms, which participants fixated on until its extinction. Due to the relationship between temporal parameters and perceived urgency of tactile cueing, the random inter-pulse interval length minimizes the possible effects of expectancy and perceived urgency of a presented signal (van Erp, 2005; Godfroy-Cooper et al., 2015). Simultaneous with the offset of the fixation cross, the target cue occurred for 100 ms at one of the 35 potential locations in a random order. Immediately following the offset of the target cue, a visual pointer appeared on the visual plane in a random location in order to maintain the desired open-loop feedback condition. Participants were instructed to move the pointer using the leg-mounted trackball to the perceived target location, then validate their response by clicking the mouse. The target was extinguished before the target pointer was introduced allowing participants to make the location determination with no visual feedback about their performance. Upon validation of the response, the trial was terminated and the next trial launched after a 1500 ms interval. Each participant completed 10 repetitions for each 35 target points for a total of 350 repetitions for each of the 3 modality conditions. In testing, repetitions were divided into two testing blocks of 175 repetitions per condition, for a total of 6 testing blocks. Participants received a 3 minute break between each testing block and a 30 second break after every 70 repetitions during each testing block. All participants experienced all experimental conditions in a random order, and each test block took roughly 30 minutes to complete. Each participant completed the experiment over two testing session.^1^

#### 2.2.5 Measures

The experiment described above is a repeated measures, within subject design with parametric data. The measures are: (i) *precision*—a measure of how close one participant's responses are to each other, measured in variance of responses, and (ii) *accuracy*—a measure of distance from the response center of gravity to the true target position. The measures of precision and accuracy were calculated for each target point in each modality condition as well as the predicted bimodal condition under the MLE model (VH_MLE_). The response data collected was in the form of *x* and *y* position coordinates of the clicked response point compared to the *x* and *y* position coordinates of the actual target, and response time for each target. Additionally, biographical and anthropometric data from each participant was collected in order to investigate the effects of body size and curvature, as well as eye and hand dominance.

Target point locations were represented by their position in degrees of visual angle in azimuth ($\phi_{a}=\{\pm 25^{{}^{\circ}},\pm 17^{{}^{\circ}},\pm 8^{{}^{\circ}},0^{{}^{\circ}}\}$) and elevation ($\phi_{e}=\left\{\pm 10^{{}^{\circ}},\pm 5^{{}^{\circ}},0^{{}^{\circ}}\right\}$). Let the position vector of the *i*^th^ targer, where *i* ∈ [1, 35], be represented by $r_{O\to T_{i}}^{⊤}=\left[x_{{\text{T}}_{i}}\;y_{{\text{T}}_{i}}\right]$. Then, target orientation is defined as:


(8) $$\begin{array}{l} \theta_{{\text{T}}_{i}}=\tan^{-1}\left(\frac{y_{{\text{T}}_{i}}}{x_{{\text{T}}_{i}}}\right) \end{array}$$

**Figure 4:**
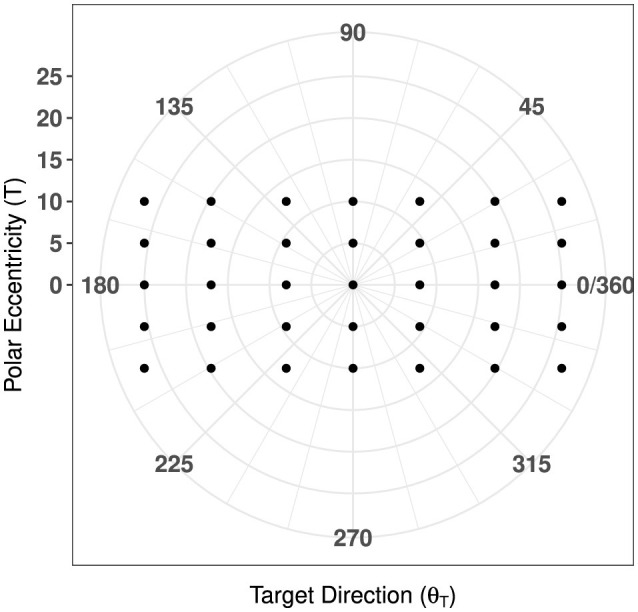
Map of target directions with respect to polar eccentricity.

The magnitude of this target vector, $r_{O\to T_{i}}={\left\|r_{O\to T_{i}}\right\|}_{2}$, was used to provide a measure of overall eccentricity for each target point in the polar coordinate plane, as visualized in Figure 4.

##### 2.2.5.1 Precision

As described above, the variance of the response in each direction around each target was computed. Since response data was collected as *x* and *y* coordinates from the screen origin *O*, variance was calculated for the responses in each *x* and *y* direction. By computing the variances in each direction separately as opposed to a single distance vector, the effects of direction (azimuth and elevation) with respect to localization precision may be compared in each modality. Under the assumption of normality, the *2D precision* is a sum of the variances in the *x* and *y* directions such that:


(9) $$\begin{array}{l} \sigma_{xy_{i,j}}^{2}=\sigma_{x_{i,j}}^{2}+\sigma_{y_{i,j}}^{2} \end{array}$$

which was calculated for each target point *i* ∈ [1, 35] and in each modality *j* ∈ {V, H, VH}. Using the variances of response components in the *x* and *y* directions, the 2D covariance matrix for each target point was determined. The covariance matrices of the form:


(10) $$\begin{array}{l} \text{Cov}\left(x_{i,j},y_{i,j}\right)=\left[\begin{array}{cc} \sigma_{x_{i,j}}^{2} & \text{Cov}\left(x_{i,j},y_{i,j}\right)\\ \text{Cov}\left(x_{i,j},y_{i,j}\right) & \sigma_{y_{i,j}}^{2} \end{array}\right] \end{array}$$

were used to construct 95% confidence ellipses illustrating response distribution around each target point, which provide a measure of dispersion and spread of the responses around their center of gravity compared to the true target position. The center of gravity is the vector $r_{O\to {\text{R}}_{i,j}}^{⊤}=\left[x_{i,j}\;y_{i,j}\right]$. The direction of the main eigenvector of the covariance matrix or, equivalently, the ellipse orientation was found as:


(11) $$\begin{array}{l} \theta_{{\text{R}}_{i,j}}=\tan^{-1}\left(\frac{\lambda_{{\text{min}}_{i,j}}}{\lambda_{{\text{max}}_{i,j}}}\right) \end{array}$$

where λ_max_*i, j*__ and λ_min_*i, j*__ are the maximum and minimum eigenvalues of the covariance matrix. It is worth noting that the ellipse orientation represents the direction of maximal dispersion for each target point. This was compared to the true target direction to produce a measure of *orientation deviation*, representing the angular difference, in degrees, between the true target direction and the direction of maximal dispersion of responses such that:


(12) $$\begin{array}{l} \Delta_{{\text{O}}_{i,j}}=|\theta_{{\text{T}}_{i}}-\theta_{{\text{R}}_{i,j}}| \end{array}$$

Finally, a measure of anisotropy, ϵ_*i, j*_, indicates the presence of a preferred direction of responses around each target point. Anisotropy was calculated as a normalized ratio of the major and minor axes of the 95% confidence ellipses such that:


(13) $$\begin{array}{l} \epsilon_{i,j}=\sqrt{1-{\left(b_{i,j}/a_{i,j}\right)}^{2}} \end{array}$$

where $a_{i,j}=\sqrt{5.991\lambda_{{\text{max}}_{i,j}}}$ represents the length of the major axis and $b_{i,j}=\sqrt{5.991\lambda_{{\text{min}}_{i,j}}}$ represents the length of the minor axis of the ellipse. As a ratio between major and minor axes, a value of ϵ_*i, j*_ close to 1 indicates no preferred direction and a value close to 0 indicates a preferred direction.

##### 2.2.5.2 Accuracy

Accuracy was computed as the euclidean distance between the response center of gravity and the true target positions for each target *i* ∈ [1, 35] and in each modality *j* ∈ {V, H, VH}, represented by the error vector $r_{{\text{T}}_{i}\to {\text{R}}_{i,j}}^{⊤}=\left[\left(x_{{\text{T}}_{i}}-x_{i,j}\right)\;\left(y_{{\text{T}}_{i}}-y_{i,j}\right)\right]$. The length of the error vector, $r_{{\text{T}}_{i}\to {\text{R}}_{i,j}}={\left\|r_{{\text{T}}_{i}\to {\text{R}}_{i,j}}\right\|}_{2}$ represents the magnitude of accuracy, with a smaller value representing more accurate responses. The positive *x* direction is pointing right, the positive *y* direction is pointing up, and the true target point is at the center of the reference frame. The direction of the center of gravity of the responses with respect to the center of the reference frame *O*, defined as:


(14) $$\begin{array}{l} \theta_{{\text{CG}}_{i,j}}=\tan^{-1}\left(\frac{y_{i,j}}{x_{i,j}}\right) \end{array}$$

was calculated and compared to the true target direction to produce a measure of *directional deviation* such that:


(15) $$\begin{array}{l} \Delta_{{\text{D}}_{i,j}}=|\theta_{{\text{T}}_{i}}-\theta_{{\text{CG}}_{i,j}}| \end{array}$$

In the following analysis, we assumed that (1) all target positions were equally likely (participants had no prior assumption regarding the number, order, and spatial configuration of the targets) and (2) the noise corrupting the visual signal was independent from the one corrupting the haptic signal (Godfroy-Cooper et al., 2015). The response data was assumed to follow a 2D normal distribution dependent on the *x* and *y* directions (Godfroy-Cooper et al., 2015) and that takes into account the direction of the distribution (van Beers et al., 1999). Under these assumptions, the 2D probability density function may be written as:


(16) $$\begin{array}{r} P\left(\delta_{x},\delta_{y}\right)=\frac{1}{2\pi }\sigma_{x}\sigma_{y}\sqrt{1-\rho^{2}}\exp\{-\frac{1}{2\left(1-\rho^{2}\right)}\left[\frac{{\left(x-\mu_{x}\right)}^{2}}{\sigma_{x}^{2}}\right]\\ +\frac{{\left(y-\mu_{y}\right)}^{2}}{\sigma_{y}^{2}}-\frac{2p\left(x-\mu_{x}\right)\left(y-\mu_{y}\right)}{\sigma_{x}\sigma_{y}}\} \end{array}$$

where μ_*x*_, μ_*y*_ are the means in the *x* and *y* directions, $\sigma_{x}^{2},\sigma_{y}^{2}$ are the variances in the *x* and *y* directions, and ρ is the correlation coefficient.

Finally, a measure of redundancy gain was used to represent the multi-sensory integration response. Redundancy gain describes the improvement in performance that is expected as a result of providing redundant information in another modality (Godfroy-Cooper et al., 2015; Ernst and Luca, 2011). Based on the hypothesis of multisensory integration, localization accuracy should increase in the multimodal condition compared to both unimodal conditions because of the presence of redundant signals. Assuming that vision is the more effective unimodal condition, the redundancy gain is provided by:


(17) $$\begin{array}{l} {\text{RG}}_{i}=100\left(\frac{\sigma_{xy_{i,\text{VH}}}^{2}}{\sigma_{xy_{i,\text{V}}}^{2}}\right) \end{array}$$

#### 2.2.6 Statistical analysis

##### 2.2.6.1 Unimodal space characterization

Each unimodal space was characterized individually to investigate trends across eccentricity in each direction. Precision and accuracy were first assessed as a function of overall target eccentricity, *T*_*i*_ and target direction, θ_*T*_*i*__ for each target *i* ∈ [1, 35]. Then, precision and accuracy were assessed as a function of the target main direction, *i.e*.azimuth and elevation separately, because of the differences in eccentricity in both directions. In each direction, measures were considered for all targets in each direction, and for those strictly on the SMP or HMP. For each measure in each direction, Pearson correlation tests were used to investigate the hypothesis of collinearity between responses in the x and y directions in each modality. Promising target groupings in each modality were determined assuming independence of each target, and Levene Tests were conducted to test for homogeneity of variance between each group. Then, one-way and two-way ANOVAs were performed to assess successively the effects of hemifields (left, right, upper, lower), and eccentricity in azimuth and elevation. Finally, simple linear regressions were used to investigate the effect and significance of eccentricity in each direction for each measure.

##### 2.2.6.2 Measuring the MLE and modality comparison

The MLE predictions for the bimodal condition for each measure of precision and accuracy were calculated according to the methods described above, which were compared to the observed unimodal and bimodal conditions, V, H, and VH. Each of the unimodal measures were applied to the MLE model to develop predictions for when performance, characterized by 2D-Precision (Equation 9) and 2D-Accuracy (Equation 4) in the bimodal condition exceeds that of the most precise modality alone. The MLE predictions were compared to the observed unimodal and bimodal conditions, V, H, and VH to verify the model. Using this information, regression and classification methods were employed to develop a model that best predicts when performance in the multimodal condition exceeds that of the most precise unimodal condition.

The same analysis as the unimodal characterization described above was conducted for the observed and predicted bimodal VH condition in order to provide a comparison between the characteristics of each. To test for possible effects of each modal condition on each measure of precision and accuracy, a univariate repeated measures analysis of variance (ANOVA) test was conducted to determine whether the differences between different independent modalities was significant or not. Specifically, the analysis focused on the differences between the observed VH condition compared to the bimodal performance predicted by the MLE model, as well as conditions under which the observed VH performance was statistically equivalent to, or exceeded that of the most reliable unimodal condition.

As described in Equation 17 redundancy gain was calculated and assessed as a function of target direction and eccentricity, as well as a function of precision from the most precise unimodal condition. The patterns of redundancy gain provide a measure of when the effect of multisensory integration, as realized through the introduction of redundant information through the haptic modality, is most helpful relative to the best unimodal condition alone. In this way, it is used as a measure of potential integration effect.

All of the effects described here were statistically significant at *p* < 0.05 or better.

## 3 Results

The experimental data are summarized in Table 1. All results of statistical analyses are contained in referenced tables located in the Appendix.

**Table 1:** Raw variable descriptions.

**Variable**	**Description**	**Type**	**Levels/range**
Target ID	Target point index	Categorical	1-35
Participant ID	Participant identification	Categorical	1–16
*X*_T_	Target x coordinate (m)	Numerical	(-0.5, 0.5)
*Y*_T_	Target y coordinate (m)	Numerical	(-0.2, 0.2)
Distance	Geometric distance between response and actual target location (m)	Numerical	(0, 0.2)
*X*_R_	Response x coordinate (m)	Numerical	(-0.7, 0.7)
*Y*_R_	Response y coordiante (m)	Numerical	(-0.4, 0.4)
*t*	Response time (s)	Numerical	(0,5)
Modality	Sensory modality condition	Categorical	V, A, H, VA, VH, AH, VAH
Azimuth	Spatial positioning location in azimuth of targets (deg)	Categorical	-25, -17, -8, 0, 8, 17, 25
Elevation	Spatial positioning locations in elevation of targets (deg)	Categorical	-10, -5, 0, 5, 10
Sex	Sex of participant	Categorical	M, F
Age	Age of participant (years)	Numerical	22–34
*WC*	Waist circumference (cm)	Numerical	72–116
*HD*	Participant hand dominance	Categorical	R, L
*ED*	Participant eye dominance	Categorical	R, L

### 3.1 Inter-individual differences

Prior to the analysis, target locations and localization responses for the female participants were scaled to fit the reference frame of the male target locations to allow for the combination and joint comparison of male and female response data. Meanwhile, because the human body was approximated as a cylinder, and that the tactor's placement could not be fitted between the participants, each participant's waist circumference was measured and used to assess it's possible effect on localization performance.

Single linear regressions were performed to identify if waist circumference, gender, age, eye- and hand-dominance were significant predictors for precision and accuracy in as a function of the modality of the target presentation and as a function of target direction. Indeed, no effect of waist circumference was expected in the unimodal V condition, and waist circumference would likely affect accuracy in azimuth (because it a wider waist circumference would bring the tactors closer to each other), but not in elevation. No effect was expected for precision. For the H modality, waist circumference was a significant predictor for accuracy [*R*^2^ = 0.01, *F*_(1, 558)_ = 0.97, *p* = 0.005], significant for targets located in azimuth [*R*^2^ = 0.17, *F*_(2, 109)_ = 11.6, *p* < 0.001], but not for targets located in elevation [*R*^2^ = 0.03, *F*_(2, 77)_ = 1.22, *p* = 0.30]. In azimuth, accuracy decreased in general with higher waist circumference, regardless of eccentricity. In elevation, conversely, as expected, waist circumference was not a significant predictor. waist circumference was not a significant predictor of precision [*R*^2^ = 0.004, *F*_(1, 552)_ = 2.58, *p* = 0.1]. In the V and VH conditions, waist circumference was not a significant predictor of accuracy [V: *R*^2^ = 0.001, *F*_(1, 552)_ = 0.6, *p* = 0.4; VH: *R*^2^ = 0.003, *F*_(1, 558)_ = 1.5, *p* = 0.16]. These results suggest that CW contributed to a localization bias in the H condition, with H targets being perceived closer to the body midline for the largest waist circumferences. However, the experimental repeated-measures design ensures that this factor is controlled for and therefore not impacting the general conclusions of this research. Meanwhile, the effects of gender, age, eye and hand dominance were also tested as a function of the target modality. No significant and consistent effect was observed. Taken altogether, these results justify the further pooling of the data. However, the question of body shape idiosyncrasies and its influence on target localization will need to be addressed in further studies.

Outliers were identified beyond 3 standard deviations based on the Mahalanobis Distance measures of *x*_R_ and *y*_R_ as two possibly correlated dependent variables. This method facilitates outlier detection of multivariate data by scaling the contribution of each variable to the distance away from the mean according to the variability of each dependent variable (Ghorbani, 2019). From each modality, there were 7.23% (V), 11.58% (H), and 6.87% (VH) outliers removed. It may be noted that the increased number of haptic outliers could be a result of individual perceptual differences that are more exaggerated than visual perceptual differences.

**Figure 5:**
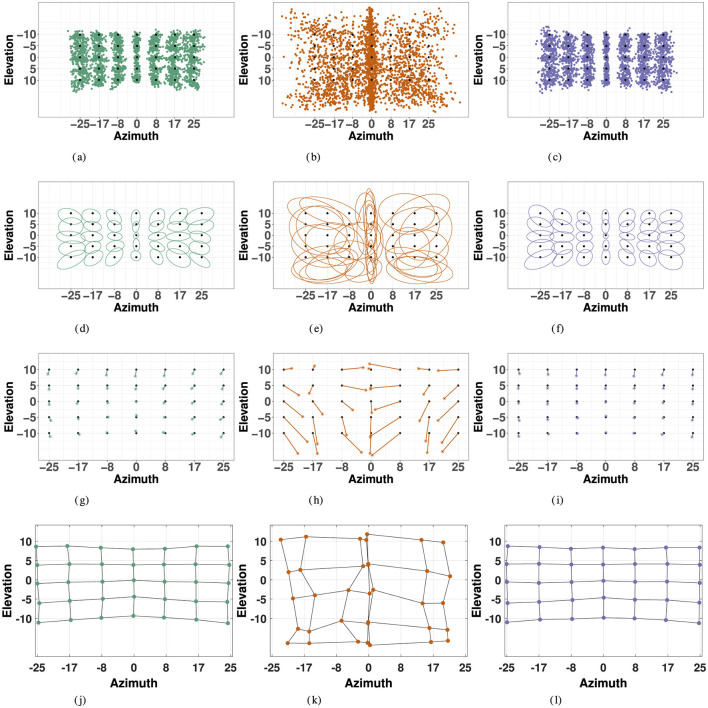
Localization responses (first row), 2D precision (second row), 2D accuracy (third row), and spatial distortion (fourth row) for the three modalities: visual (left), haptic (middle) and combined visual-haptic (right). 2D precision is represented by 95% confidence ellipses constructed from eigenvectors of covariance matrices from responses in each modality. 2D accuracy is represented by error vectors connecting the true target point to the response center of gravity, marked by the *. Distortion plots were constructed by horizontally and vertically connecting the center of gravity of responses for each consecutive target point, and illustrate compression of space in each modality. **(A)** Visual Responses. **(B)** Haptic Responses. **(C)** VH Responses. **(D)** Visual Precision. **(E)** Haptic Precision. **(F)** VH Precision. **(G)** Visual Accuracy. **(H)** Haptic Accuracy. **(I)** VH Accuracy. **(J)** Visual Distortion. **(K)** Haptic Distortion. **(L)** VH Distortion.

Figures 5A–C display the responses compared to the actual target locations in the each modality for all 35 target points. The variables of interest, listed in Table 2 were computed according to the methods previously described, and observed measures for precision, accuracy, and distortion are visualized for each modality are visualized in Figure 5.

**Table 2:** Computed variable descriptions.

**Variable**	**Description**	**Equation**	**Range/units**
L/R	Indicates whether a point is positioned in the left (L), right (R) hemisphere, or on the SMP	-	L, R, SMP
U/D	Indicates whether a point is positioned in the upper (U), lower (D) hemisphere, or on the HMP	-	U, D, HMP
*r*_*O*→_*T*__*i*__	Magnitude of target vector; measure of polar eccentricity	$r_{O\to T_{i}}=\sqrt{x_{{\text{T}}_{i}}^{2}+y_{{\text{T}}_{i}}^{2}}$	[m]
*x*_*i, j*_	Center of gravity of responses in the *x* direction	$x_{i,j}=\frac{\sum R_{x}}{n_{R}}$	(-0.7, 0.7) [m]
*y*_*i, j*_	Center of gravity of responses in the *y* direction	$y_{i,j}=\frac{\sum R_{y}}{n_{R}}$	(-0.4, 0.4) [m]
$\sigma_{xy}^{2}$	2D Precision	$\sigma_{xy_{i,j}}^{2}=\sigma_{x_{i,j}}^{2}+\sigma_{y_{i,j}}^{2}$	Range
θ_T_	Target orientation	$\theta_{{\text{T}}_{i}}=\tan^{-1}\left(\frac{y_{{\text{T}}_{i}}}{x_{{\text{T}}_{i}}}\right)$	0°-360°
θ_R_	Direction of maximal dispersion	$\theta_{{\text{R}}_{i,j}}=\tan^{-1}\left(\frac{\lambda_{{\text{min}}_{i,j}}}{\lambda_{{\text{max}}_{i,j}}}\right)$	0°-360°
Δ_O_	Orientation deviation	Δ_O_*i*__ = |θ_T_*i*__ − θ_R_*i, j*__|	0°-360°
ϵ	Measure of anisotropy	$\epsilon_{i,j}=\sqrt{1-{\left(b_{i,j}/a_{i,j}\right)}^{2}}$	0-1
*r*_T_*i*_ → R_*i, j*__	2D Accuracy	$r_{{\text{T}}_{i}\to {\text{R}}_{i,j}}=\sqrt{{\left(x_{{\text{T}}_{i}}-x_{{\text{R}}_{i,j}}\right)}^{2}+{\left(y_{{\text{T}}_{i}}-y_{{\text{R}}_{i,j}}\right)}^{2}}$	(0, 0.2) [m]
θ_CG_	Orientation of response center of gravity	$\theta_{{\text{CG}}_{i,j}}=\tan^{-1}\left(\frac{y_{i,j}}{x_{i,j}}\right)$	0°-360°
Δ_D_	Direction deviation	Δ_D_*i*__ = |θ_T_*i*__ − θ_CG_*i, j*__|	0°-360°

### 3.2 Visual space characterization

#### 3.2.1 Precision

**Figure 6:**
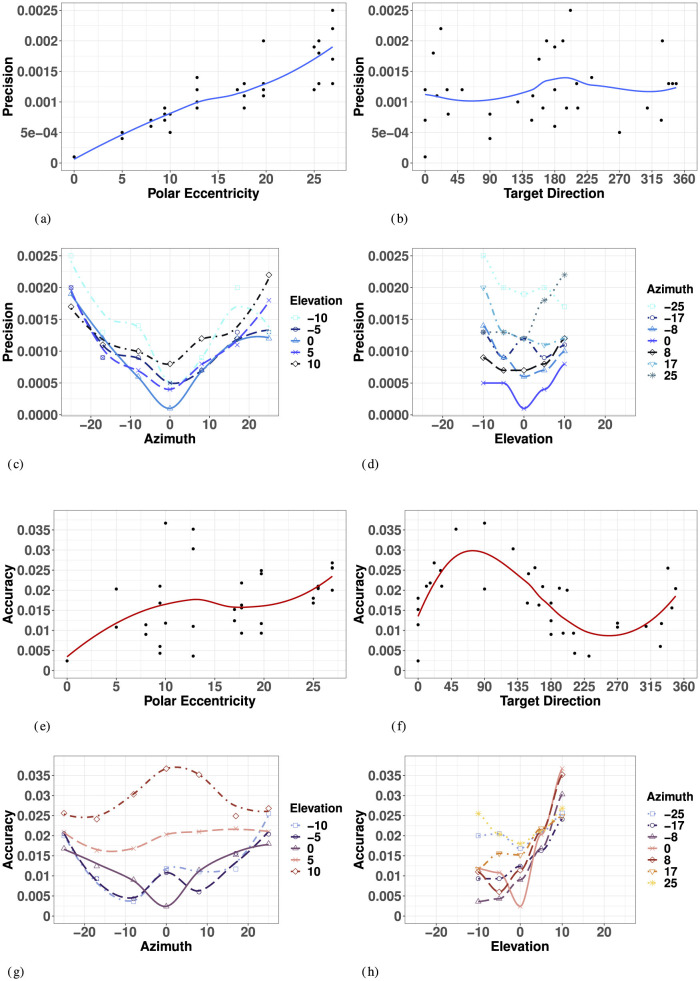
Effect of target eccentricity on VISUAL 2D precision **(A–D)** and accuracy **(E–H)** in the polar **(A, B, E, F)** and cartesian **(B, C, G, H)** coordinate systems. Note that all points are fit using a locally estimated scatterplot smoothing (LOESS) function. **(A)** V 2D Precision as a function of polar eccentricity. **(B)** V 2D Precision as a function of target direction. **(C)** V 2D Precision as a function of eccentricity in azimuth for all elevations. **(D)** V 2D Precision as a function of eccentricity in elevation for all azimuths. **(E)** V 2D Accuracy as a function of polar eccentricity. **(F)** V 2D Accuracy as a function of target direction. **(G)** V 2D Accuracy as a function of eccentricity in azimuth for all elevations. **(H)** V 2D Precision as a function of eccentricity in elevation for all azimuths.

Overall, visual precision follows a radial pattern characterized by a systematic decrease with eccentricity in the polar coordinate system. Figure 5 illustrates a consistent trend of inward ellipse orientation and increasing variable error with polar eccentricity [*F*_(11, 23)_ = 7.58, *p* < 0.001]. The variance ellipses are mostly aligned in the direction of the targets relative to the initial fixation point. These scatter properties are consistent with the polar organization of the visuomotor system (van Opstal and van Gisbergen, 1989). Figure 6A indicates that visual 2D precision decreases almost linearly with eccentricity, suggesting no strong effect of target direction. Indeed, as illustrated in Figure 6B the effect of target direction in the polar coordinate system was not significant [*F*_(27, 7)_ = 1.29, *p* = 0.39]. Note that the overall 2D precision was best for targets with a 90°/270° orientation and the lowest for targets with a 0°/180°/360° orientation, an effect of the differences in eccentricities tested in elevation (up to 10°) and in azimuth (up to 25°).

In azimuth (for all elevations), as seen in Figure 6C and Appendix Table 1, visual localization was significantly more precise for targets on the SMP than in the periphery (left/right). There were no significant differences between left and right hemifield when considering values of azimuth for all elevations, nor when considering strictly the targets on the HMP.

In elevation (for all azimuths), as seen in Figure 6D and Appendix Table 1, there was no significant effect of hemifield, and 2D precision was statistically equivalent for upper hemifield, lower hemifield and HMP. When considering strictly the targets in the SMP, there was still no effect of hemifield. With symmetry over the HMP and SMP, the true effects of eccentricity on visual precision may be assessed using absolute rather than signed values of eccentricity for both directions of azimuth and elevation.

A two-way ANOVA with absolute eccentricity in azimuth and absolute eccentricity in elevation as fixed factors showed that visual precision decreased with eccentricity in both azimuth and elevation, with no significant interaction between the two directions. Results of the ANOVA are listed in Appendix Table 3. As a consequence, the lowest precision is observed for the more peripheral targets. Note that the differences in p-values for azimuth and elevation are partly due to the magnitude and levels of the eccentricities tested in the two orthogonal directions.

#### 3.2.2 Accuracy

Visual accuracy was characterized mostly by a systematic undershoot of the responses, *i.e*., the error vector's direction was opposite to the direction of the target in the polar coordinate system, as seen in Figure 5, resulting in a compression of the perceptive space more pronounced in the vertical direction.

Overall, the 2D accuracy, as characterized by the length of the error vectors, increased with polar eccentricity as seen in Figure 6E, though the effect did not reach significance [*F*_(11, 23)_ = 1.15, *p* = 0.37]. The overall effect of target direction in the polar coordinate system was also not significant [*F*_(11, 23)_ = 1.79, *p* = 0.22], but one can see from Figure 6F that accuracy seems to be different as a function of the orthogonal azimuth and elevation axes.

In azimuth, as seen in Figure 6G and Appendix Table 1, there were no significant differences between left and right hemifield or the SMP on visual 2D accuracy when considering targets in all elevations, nor when considering strictly the targets in the HMP. With symmetry over the SMP, visual accuracy may be assessed in terms of absolute eccentricity in azimuth.

In elevation, as seen in Figure 6H and Appendix Table 1, 2D visual localization was significantly more accurate in the lower than in the upper hemifield, an effect previously reported in the literature (Abrams et al., 2012). Interestingly, 2D accuracy in the HMP was statistically equivalent to that in the lower hemifield. Because of the lack of symmetry around the HMP, visual accuracy must be assessed in terms of signed rather than absolute values of eccentricity in elevation.

An ANOVA with absolute eccentricity in azimuth and signed eccentricity in elevation as fixed factors showed that visual 2D accuracy decreased with eccentricity in both azimuth and elevation, and that a significant interaction was present between the two directions. Results of the ANOVA are listed in Appendix Table 3. Visual accuracy in azimuth significantly decreased with increasing values of eccentricity for targets located in the lower hemifield and in the HMP, but not for targets located in the upper hemifield (see Figure 6G, elevations 5° and 10°). In elevation, the effect of eccentricity was significant only in the upper hemifield, with particularly significant differences occurring at 5° and 10° in elevation, leading to an asymmetrical compression of space in elevation, mostly on the SMP, with a more pronounced effect in the upper hemifield.

### 3.3 Haptic space characterization

#### 3.3.1 Precision

**Figure 7:**
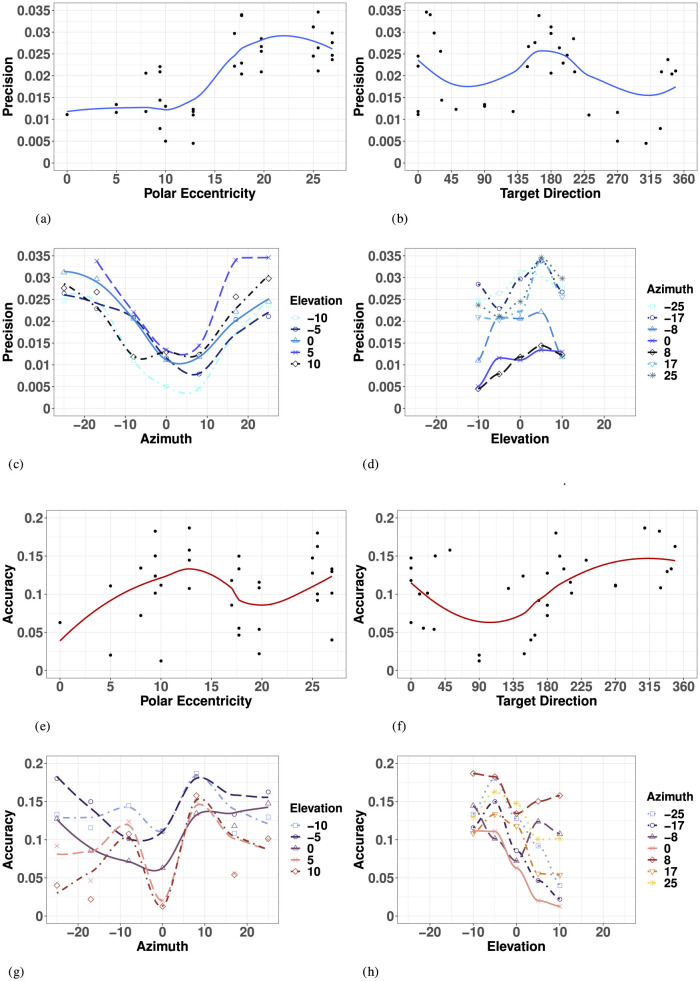
Effect of target eccentricity on HAPTIC 2D precision **(A–D)** and accuracy **(E–H)** in the polar **(A, B, E, F)** and cartesian **(B, C, G, H)** coordinate systems. Note that all points are fit using a LOESS function. **(A)** H 2D Precision as a function of polar eccentricity. **(B)** H 2D Precision as a function of target direction. **(C)** H 2D Precision as a function of eccentricity in azimuth for all elevations. **(D)** H 2D Precision as a function of eccentricity in elevation for all azimuths. **(E)** H 2D Accuracy as a function of polar eccentricity. **(F)** H 2D Accuracy as a function of target direction. **(G)** H 2D Accuracy as a function of eccentricity in azimuth for all elevations. **(H)** H 2D Precision as a function of eccentricity in elevation for all azimuths.

Overall, haptic 2D precision exhibited strong symmetry along the SMP, with no evidence for a “preferred” elevation. The variance ellipses were mostly aligned in the direction of the targets relative to the initial fixation point, with a greater fit in the SMP than in the left or right hemifields. Haptic 2D precision generally decreased with polar eccentricity [*F*_(11, 23)_ = 6.42, *p* < 0.001], as seen in Figure 7A. However, it did not vary systematically as a function of target direction [*F*_(27, 7)_ = 2.64, *p* = 0.09], as seen in Figure 7B, suggesting differences in precision along the two orthogonal axes, azimuth and elevation.

In azimuth (for all elevations), as seen in Figure 7C and Appendix Table 4, haptic localization was significantly more precise at the center (SMP) than in the periphery (left/right). There were no significant differences between left and right hemifields when considering all elevations, nor when considering strictly the targets in the HMP.

Likewise, as seen in Figure 7D and Appendix Table 4, there was no significant effect of upper and lower hemifield when considering elevation for all azimuths, nor when considering strictly the targets in the SMP. Note that in elevation, there was also no significant difference between hemifield (either upper or lower) and the SMP. With symmetry over the SMP, the true effect of eccentricity with respect to haptic precision can be assessed using absolute rather than signed eccentricity values for both azimuth and elevation.

A two-way ANOVA with absolute eccentricity in azimuth and absolute eccentricity in elevation as fixed factors indicated a significant effect of eccentricity in azimuth, but not in elevation or in the interaction between the two. Results of the ANOVA are listed in Appendix Table 6. While we see a significant decrease in precision with eccentricity in azimuth for all elevations, this effect is not quite linear, as illustrated by Figure 7C. Indeed, there was no significant difference in precision between absolute eccentricity values of 0° and 8° azimuth (for all elevations), nor between values of 17° and 25° azimuth (for all elevations). However, the difference between 8° and 17° was significant indicating a statistically significant decrease in precision as eccentricity increased only beyond 8° absolute eccentricity in azimuth. Although there was no significant effect of eccentricity in elevation on haptic precision, the best precision is achieved around −10° of eccentricity and the lowest precision for +5° degrees of eccentricity.

#### 3.3.2 Accuracy

Overall, haptic localization 2D accuracy was characterized by a nonlinear, but systematically grouped pattern of performance across the haptic space. Haptic localization 2D accuracy did not decrease linearly as a function of eccentricity, suggesting an effect of target direction and that the representation of the haptic space was not encoded in a polar coordinate system like vision. Indeed, there was no significant effect of overall eccentricity on haptic accuracy [*F*_(11, 23)_ = 1.421, *p* = 0.23], as illustrated in Figure 7E. Likewise, as illustrated by Figure 7F, haptic accuracy was sensitive to target direction, though the effect did not reach significance [*F*_(27, 7)_ = 3.046, *p* = 0.0658]. 2D haptic accuracy was the highest for targets oriented at 90° (upper SMP) and the least accurate in the opposite direction.

In azimuth (for all elevations), as seen in Figure 7G and Appendix Table 4, there was no significant difference in 2D haptic accuracy between the left and right hemifields or the SMP, nor when considering targets strictly in azimuth. Therefore, the true effect of eccentricity in azimuth can be assessed by using absolute rather than signed values of eccentricity.

Conversely, 2D haptic localization was significantly more accurate in the upper than in the lower hemifield, as illustrated in Figure 7H and Appendix Table 4. However, there was no significant difference in 2D Haptic accuracy in the HMP between either hemifield, suggesting an absence of a distinct haptic horizon.

An ANOVA with absolute eccentricity in azimuth and signed eccentricity in elevation as fixed factors showed that target eccentricity in azimuth significantly modifies 2D Haptic accuracy. Results of the ANOVA are listed in Appendix Table 6. Unlike for vision, 2D Haptic accuracy did not decrease quite linearly with eccentricity. Indeed, comparing specific groupings revealed no significant difference between eccentricities of 0° and 17° to each other, and 8° and 25° to each other, but did identify a significant difference between the two groups (0° and 17°) and (8° and 25°). Results from van Erp (2005) suggest there are at least two torso-based egocenters. With the three significant groupings identified, our results verify the existence of midlines on each left and right coronal plane, as well as a distinct center midline. These results corroborate the existence of distinct egocenters reported in the literature, reportedly positioned approximately 3 cm to the left and right of participants' midline on the coronal plane (a vertical plane running from side to side; divides the body or any of its parts into anterior and posterior portions), and could account for the difference in directional bias reported in the proximal localization study in Choleiwak et al. (2004) and in the distal localization study of van Erp (2005).

### 3.4 Applying the MLE model: observed vs. predicted bimodal visual-haptic performance

#### 3.4.1 Bimodal visual-haptic 2D precision

The MLE model predicted that bimodal precision would be at least as precise as the most reliable unimodal condition, with an improved performance in regions where the reliability of the unimodal condition is lower. With vision significantly more precise than haptic for the entire space considered, it was given a much higher weight (recall Equation 3a-b), resulting in VH predictions (Equation 2) that share many characteristics with the unimodal V space.

**Figure 8:**
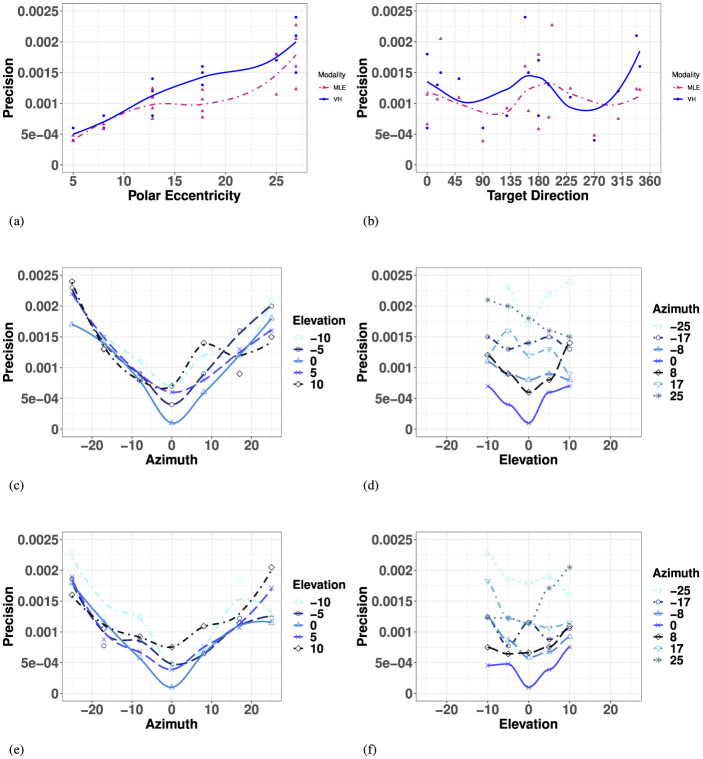
Effect of target eccentricity and direction **(A, B)** on OBSERVED VH **(C, D)** vs. PREDICTED MLE **(E, F)** 2D precision in the polar **(A, B)** and cartesian **(B–E)** coordinate systems. Note that all points are fit using a LOESS function. **(A)** OBSERVED VH vs. MLE 2D Precision as a function of polar eccentricity. **(B)** OBSERVED VH vs. MLE 2D Precision as a function of target direction. **(C)** OBSERVED VH 2D Precision as a function of eccentricity in azimuth for all elevations. **(D)** OBSERVED VH 2D Precision as a function of eccentricity in elevation for all azimuths. **(E)** PREDICTED MLE 2D Precision as a function of eccentricity in azimuth for all elevations. **(F)** PREDICTED MLE 2D Precision as a function of eccentricity in elevation for all azimuths.

The model predicted a significant decrease in precision with overall eccentricity [*F*_(11, 23)_ = 26.56, *p* < 0.001], with no significant effect of target direction [*F*_(27, 7)_ = 1.26, *p* = 0.40], as visualized in Figures 8A, B. Similar to the visual modality, and illustrated in Figure 8E and listed in Appendix Table 10, the model predicted no significant difference between the left and right hemifields but did identify a significant difference between targets strictly on the SMP compared to either hemifield, left and right. In elevation (for all azimuths), as seen in Figure 8E, the model predicted no significant differences between upper and lower hemisphere, nor with the HMP. Finally, when investigating the effect of absolute eccentricity in each direction, the model predicted a significant decrease in precision with eccentricity in both azimuth and elevation, and no significant interaction between the two directions (Appendix Table 9; Figure 8F).

Overall, performance of observed bimodal 2D Precision demonstrated similar trends to that of the visual modality. Visual-haptic 2D precision also exhibited a radial pattern characterized by a systematic decrease with eccentricity, also with no significant effect of target main direction (Appendix Table 8). Figure 8A indicates that visual-haptic 2D precision decreases almost linearly with eccentricity until more extreme values, a trend that is observed for eccentricity in all target directions [*F*_(11, 23)_ = 12.7, *p* < 0.001]. Indeed, the effect of target direction in the polar coordinate system was not significant [*F*_(27, 7)_ = 1.28].

In azimuth (for all elevations), as seen in Figure 8C and listed in Appendix Table 7, VH localization was significantly more precise at the center (SMP) than in the periphery (L/R), an effect also observed in the unimodal visual and haptic modalities. There were no significant differences between left and right hemifields. In elevation (for all azimuths), as seen in Figure 8D and Appendix Table 7, bimodal 2D precision was statistically equivalent between the upper hemifield, lower hemifield, and HMP. When considering targets strictly in elevation, there is still no effect of hemifield.

An ANOVA with absolute eccentricity in azimuth and absolute eccentricity in elevation as fixed factors showed that visual precision decreased with eccentricity in both azimuth and elevation, with no significant interaction between the two directions. Results of the ANOVA are listed in Appendix Table 9. For observed bimodal 2D precision, there was a significant overall effect of eccentricity in azimuth (for all elevations). However, there was no significant effect of eccentricity in elevation (for all azimuths), an observed outcome different from the visual modality. There was no significant interaction observed between the two directions.

#### 3.4.2 Bimodal VH 2D accuracy

The MLE model predicted bimodal accuracy Equation 4 as a weighted linear sum of accuracy from each unimodal condition based on the reliability of the source, resulting in predicted accuracy that would be compromised in favor of the most precise condition (V).

**Figure 9:**
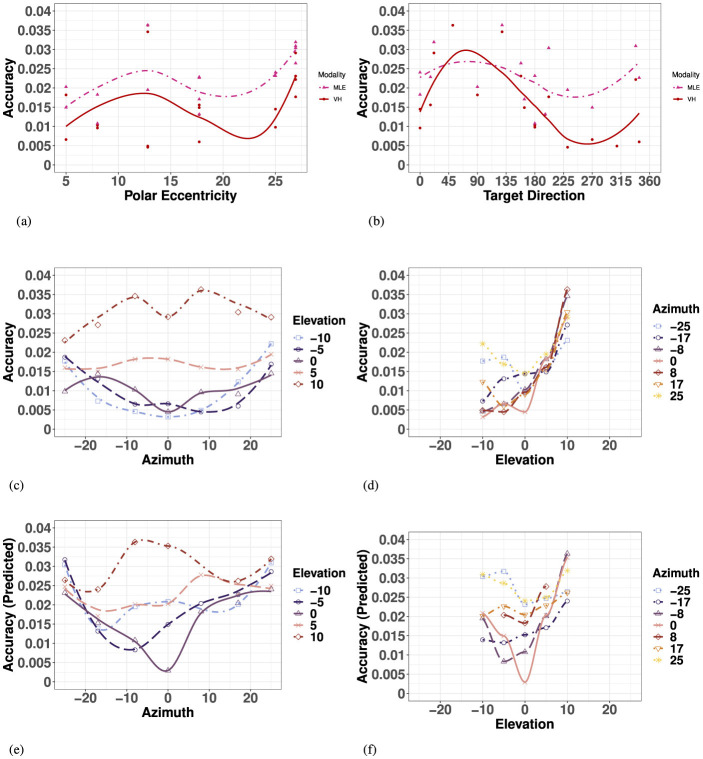
Effect of target eccentricity and direction **(A, B)** on OBSERVED VH **(C, D)** vs. PREDICTED MLE **(E, F)** 2D accuracy in the polar **(A, B)** and cartesian **(B–E)** coordinate systems. Note that all points are fit using a LOESS function. **(A)** Observed VH vs. MLE 2D Accuracy as a function of polar eccentricity. **(B)** bserved VH vs. MLE 2D Accuracy as a function of target direction. **(C)** OBSERVED VH 2D Accuracy as a function of eccentricity in azimuth for all elevations. **(D)** OBSERVED VH 2D Accuracy as a function of eccentricity in elevation for all azimuths. **(E)** PREDICTED MLE 2D Accuracy as a function of eccentricity in azimuth for all elevations. **(F)** PREDICTED MLE 2D Accuracy as a function of eccentricity in elevation for all azimuths.

The model predicted that bimodal accuracy would decrease as a function of eccentricity with no preference for target direction, as seen in Figure 8E and Appendix Table 8. Similar to the visual modality and observed VH modality, the model predicted no significant effect of left and right hemifield or the SMP (Appendix Table 7). Unlike the observed V and VH conditions, the model predicted no significant effect between upper and lower hemifields, but did predict a significant difference between accuracy directly on the HMP and the upper hemifield specifically, as seen in Figures 9B, E. Finally, the model predicted a significant decrease in bimodal accuracy with absolute eccentricity in azimuth and with signed eccentricity in elevation, but no significant interaction between the two directions (Appendix Table 9).

Overall, similar to the visual modality, performance of observed bimodal VH accuracy was characterized by a systematic undershoot of the responses resulting in a compression of space more pronounced in the vertical direction. As seen in Figure 9A and Appendix Table 8, there was no significant increase in 2D accuracy with polar eccentricity [*F*_(11, 23)_ = 7.55, *p* = 0.003], but the overall effect of target direction was significant [*F*_(27, 7)_ = 5.31, *p* = 0.014]. Similar to the visual condition, accuracy appears to be a function of the orthogonal azimuth and elevation axes.

In azimuth, as seen in Figure 9C and Appendix Table 7, there were no significant differences between left and right hemifield on bimodal 2D accuracy when considering targets of all elevations, nor when considering strictly the targets in the HMP. In elevation (for all azimuths), as seen in Figure 9D and Appendix Table 7, observed bimodal accuracy was significantly more accurate in the lower than in the upper hemisphere. Likewise, similar to the visual modality, 2D accuracy in the HMP was statistically equivalent to that in the lower hemifield, emphasizing the pronounced difference observed in the positive vertical direction (Figure 9F).

Finally, an ANOVA with absolute eccentricity in azimuth and signed eccentricity in elevation revealed that observed bimodal 2D accuracy decreased with eccentricity in both azimuth and elevation, and identified a significant effect of interaction between the two directions (Appendix Table 9).

### 3.5 Modality comparison

Repeated measures ANOVAs were conducted to compare performance metric between each modality condition. Descriptive characteristics of each condition are listed in Table 3.

**Table 3:** Characteristics of observed V, H, VH, and MLE measures of localization precision and accuracy (μ:= mean and σ:= standard deviation).

	**V**	**H**	**VH**	**MLE**
	**μ (σ)**	**μ (σ)**	**μ (σ)**	**μ (σ)**
Precision (*m*^2^)	0.00118 (5.56e-04)	0.02099 (8.73e-03)	0.00131 (6.14e-04)	0.00110 (1.10e-04)
Accuracy (m)	0.0173 (8.44e-03)	0.1081 (4.69e-02)	0.0156 (9.03e-03)	0.0233 (8.91e-03)

Note for all measures, a smaller value indicates “better” performance.

For all measures of precision and accuracy, the haptic modality performed significantly worse than V, VH, and predicted MLE. Since the MLE model aims to predict when the bimodal condition exceeds that of the more precise modality, the haptic condition was omitted from the following analysis in order to concentrate on differences between observed and predicted bimodal performance with V, which always performed better than H in the space being considered.

#### 3.5.1 Precision

**Figure 10:**
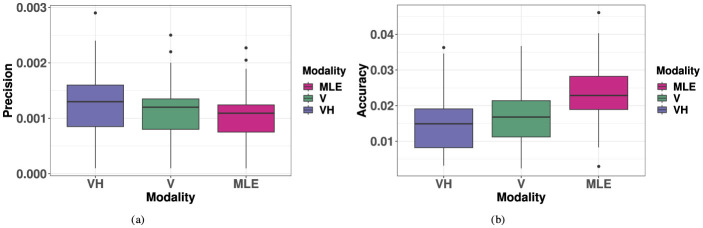
Boxplots of observed VH, V, and MLE measures of 2D localization precision and accuracy. Note for all measures, a smaller value indicates “better” performance. **(A)** 2D Precision ($\sigma_{xy}^{2}$). **(B)** 2D Accuracy (*r*).

As illustrated in Figure 10A, the MLE model predicted improved 2D precision compared to the unimodal V condition, an effect that was not observed. Comparing within each target ID, a repeated measures ANOVA with V, VH and MLE precision as fixed factors showed that the model predicted a significant improvement of precision when V and H are combined (improvement in precision for bimodal relative to the best unimodal condition) (V, MLE: *t* = 8.19, *p* < 0.001). However, there was no significant difference in precision in the observed VH condition compared to V (VH, V: *t* = 2.13, *p* = 0.12) and observed VH precision was significantly lower than predicted by the model (VH, MLE: *t* = 3.41, *p* = 0.005). Complete results of the ANOVAs are listed in Appendix Table 10.

#### 3.5.2 Accuracy

As seen in Figure 10B, the MLE model predicts that accuracy in localizing the bimodal VH targets would represent a compromise between haptic and visual performance in favor of the most precise modality, here, vision. Again, comparing within each target ID, a repeated measures ANOVA with V, VH and MLE precision as factors showed that indeed, the predicted VH localization was significantly less accurate than V localization (MLE, V: *t* = 6.16, *p* < 0.001). However, the observed bimodal accuracy was found to be, at least locally, equivalent to or to exceed that of the best unimodal condition (VH, V: *t* = −2.63, *p* = 0.038), a phenomenon unpredicted by the model. Indeed, this difference between observed VH and MLE was statistically significant (VH, MLE: *t* = −6.06, *p* < 0.001). Complete results of the ANOVAs are listed in Appendix Table 10.

Specifically, VH accuracy exceeded that of the unimodal visual condition at more extreme eccentricities in azimuth. This effect is visualized in Figure 5 accuracy and distortion plots for V and VH. In Figure 5 accuracy plots, one may notice at absolute eccentricity of 25° in azimuth, that the error vector has a stronger inward direction in the unimodal visual condition than in the VH condition. This effect is further illustrated by the distortion plot in Figure 5, that shows a stronger inward compression of space at absolute eccentricity of 25° in azimuth for V than for VH. Indeed, this effect is statistically significant (V, VH: *t* = 3.67, *p* = 0.015), as VH accuracy exceeds that of the unimodal condition at absolute eccentricity of 25° in azimuth. VH and V accuracy was statistically equivalent at all other absolute eccentricity values in azimuth (for all elevations) and for all eccentricity values in elevation (for all azimuths). It is important to note that in the visual condition alone, accuracy significantly decreased with eccentricity in azimuth, an effect that was significantly minimized at the most extreme eccentricity in the VH condition. On the contrary, this effect was not observed for specific eccentricities in elevation, with no significant differences between V and VH, a discrepancy that was likely a result of the differences in magnitudes being tested in each direction.

#### 3.5.3 Redundancy gain

**Figure 11:**
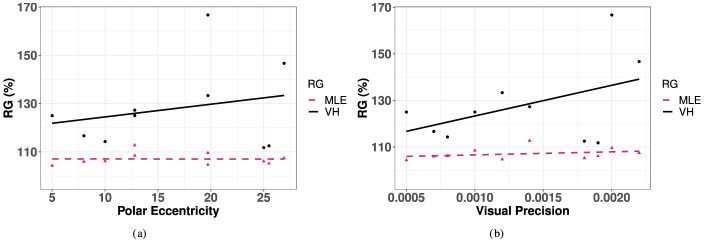
Redundancy gain for as a function of polar eccentricity and visual precision for observed VH and predicted MLE. **(A)** Polar eccentricity. **(B)** Visual precision.

The observed redundancy gain (RG) was positive for 28.6% (*i.e*., 10) of the targets tested. For these targets, there is a slight upward trend indicating an increase in redundancy gain with polar eccentricity, although this effect was not statistically significant [*R*^2^ = 0.04, *F*_(1, 8)_ = 0.33, *p* = 0.58]. Similarly, there was no statistically significant effect for this observed trend in azimuth or eccentricity [Azimuth: *F*_(3, 15)_ = 0.227, *p* = 0.876, elevation: *F*_(4, 14)_ = 0.0749, *p* = 0.56]. Finally, in order to investigate the association between RD and unimodal precision, we correlated RG with the best unimodal condition, V, as seen in Figure 11A. As visual precision decreases, it is expected that redundancy gain increases as the contribution from the redundant information in the bimodal condition becomes more important. This effect was observed in the significantly increased performance of observed VH compared to V accuracy at the most extreme values of eccentricity in azimuth, where unimodal V accuracy was the most impaired. This positive trend is observed in Figure 11B, although the effect did not reach significance.

### 3.6 Relationships between precision and accuracy

Recall that the MLE model was based on a Bayesian theory that developed predictions for 2D precision (Equation 2) according to the contribution of precision from each unimodal source. In this way, the MLE predicted precision is a direct function of visual and haptic precision. In reality, however, results of VH precision had only a statistically significant contribution from visual precision (VH, VP: *t* = 5.839, *p* = 0.001, *R*^2^ = 0.67). However, it turns out that when considering all values of each unimodal precision and accuracy, visual and haptic precision and haptic accuracy were significant predictors, while visual accuracy was not (*p* < 0.001, *R*^2^ = 0.704).

The MLE model developed predictions for 2D bimodal accuracy (Equation 4) based on a weighted combination of accuracy from each unimodal condition. Since the weights were calculated as a function of precision of each unimodal condition, the MLE model predicted a significant contribution of the visual weight, which was larger than the haptic weight for every target point (linear regression: *R*^2^ = 0.21; MLE, Wv: *t* = −4.31, *p* < 0.001). However, this trend was not observed by the true VH condition, where neither visual nor haptic weights had a statistically significant contribution. Note that the contributions of each weight provides a direct measure of contribution from precision, as they were used to calculate the weight for each source. Hence, when considering all unimodal measures of precision and accuracy, the MLE accuracy predicted a significant contribution from the visual weight, and both visual and haptic accuracy (*R*^2^ = 0.98, *p* < 0.001). However, the observed VH accuracy realized only a significant contribution from visual accuracy (linear regression: *R*^2^ = 0.81; VA accuracy, V accuracy: *t* = 11.28, *p* < 0.001), but not from visual or haptic precision. Likewise, with no significant contribution of haptic accuracy, the predicted bias from the less accurate H source was not observed, and the VH accuracy was closer to V accuracy, locally showing enhancement rather than compromise.

## 4 Discussion

### 4.1 Understanding the visual and haptic perceptive fields

Results of the present research reaffirm and extend previous sensory integration results by establishing characteristics of absolute localization performance in the 2D frontal peri-space with respect to the integration of visual and haptic spatially and temporally congruent sensory stimuli. Results of unimodal visual localization performance validated the well known characteristics of visual perception in the frontal field: visual perception is not uniform in different regions of the frontal field and precision and accuracy decrease in general with eccentricity (Levine and McAnany, 2005).

For accuracy, it is well-documented that a brief visual stimulus flashed just before a saccade is mislocalized and systematically displaced toward the saccadic landing point (Honda, 1991). This results in a symmetrical compression of visual space (Ross et al., 1997) known as “foveal bias” (Mateeff and Gourevich, 1983; Muessler et al., 1999; Kerzel, 2002) that has been attributed to an oculomotor signal that transiently influences visual processing (Richard et al., 2011). Visual space compression was also observed in perceptual judgment tasks, where memory delays were involved, revealing that the systematic target mislocalization closer to the center of gaze was independent of eye movements, therefore demonstrating that the effect was perceptual rather than sensorimotor (Sheth and Shimojo, 2001). This is clearly what the present results confirmed in the V condition. Localization performance was also more accurate in the lower visual hemifield than in the upper visual hemifield, a result already reported in the literature and referred to as vertical meridian asymmetry (Abrams et al., 2012).

Results of the unimodal haptic localization performance confirm the existence of two egocenters divided by the cutaneous region on the front of the torso (van Erp, 2005). In terms of localization, the front of the torso may be interpreted as different body parts, each having its own internal reference point. Similarly, the haptic midline down the SMP, or cutaneous region, presents characteristics of its own (body mid-axis as an internal reference point). Results of localization in terms of haptic precision indicated that precision was statistically equivalent from the midline to about ±8° in azimuth, an effect that was significant for all elevations. Likewise, there was no observed statistically significant difference between 0° and 8° degrees in azimuth, indicating that the response to a stimulus on the observer's midline is a direction that relates neither to the left nor to the right reference point. This result are in agreement with results showing that tactile stimuli presented in the cutaneous region (*i.e*., from the torso midline within a band of about 6 cm width) are represented bilaterally in the first somatic sensory cortex (Fabri et al., 2005).

For accuracy, a systematic response bias toward the midline was observed, less pronounced at the for targets located along the SMP and ±17° from the SMP. In the H condition, which involved some remapping of the tactile events from skin to external space, visual attention was not directly directed toward the locus of the H stimulation, but to the visual space where the response had to be produced. Therefore, one might expect a similar foveal bias to occur, possibly mediated by the inherent properties of the tactile receptive fields and the tactile frame(s) of reference.

By replicating existing results, the results for the unimodal visual and haptic conditions therefore provided a strong baseline for the investigation of haptic and visual-haptic performance. First, it was observed that as expected, visual localization was both more accurate and more precise than haptic localization everywhere in the investigated space. As a consequence, the visual cues were always more reliable than the haptic cues, and no significant improvement in precision was observed when the two modalities were combined. The MLE predicted the bimodal VH accuracy to be the outcome of the reciprocal of visual and haptic biases, and therefore that VH accuracy would be intermediate between V and H accuracy (*i.e*., that VH localization would be less accurate than V alone due to bias from the less reliable H source). This is not what was observed, and VH localization was mostly statistically equivalent to or more accurate than V localization alone.

Specifically, observed VH accuracy was significantly better than accuracy from V alone for targets in ±25 deg azimuth. Based on MIL-STD-1472(H) (Anon, 2020), normal line of sight is characterized by a 30° cone, inside which signals can be seen and reached without delay. Thus, the line between central and ambient field of regard occurs at ±15° in both azimuth and elevation. Thus, targets at ±25° were the only targets presented that were actually located in the accepted periphery rather than direct line of sight, essentially imposing an inherent degraded visual condition for these signals. Based on previous studies on multisensory integration, it is expected that the contribution of a redundant sensory cue will have the greatest effect when the primary sensory cue is degraded or impaired (van Erp, 2005; Godfroy-Cooper et al., 2015), which is exactly reflected in these results. Moreover, the potential for combined VH cueing to correct perceptual deficiencies occurring in the extremities of the visual field poses opportunities to improve the accessibility of information in highly dynamic environments, or extend the limits of current design constraints (Anon, 1997).

### 4.2 Limitations of the current study

The current study was somewhat constrained by limitations of the equipment used in testing. Recognizing these constraints, the following results should be addressed:

- Differences in magnitude of eccentricity tested in each direction. The visual target points were scaled to fit the size of the screen used in the experiment. Because of this, effects of eccentricity had to be assessed in azimuth and elevation separately, rather than grouped eccentricity.
- Range of eccentricities tested. The entire visual field was mostly contained in the direct field of regard, possibly masking effects that occur beyond this boundary. For instance, a significant improvement was observed between V and VH accuracy only at ±25° in azimuth, but the largest magnitude in elevation that was assessed was ±10°. Thus, conclusions about the effect in the vertical direction could not truly be assessed against those in the horizontal direction. Without expanding the size of the visual field used in testing, this limitation may also be overcome by imposing a degraded visual stimulus to modify the reliability of the signal.
- Number and placement of tactors on suit. A universally sized vest with fixed tactor positions was used for all participants. Thus, the tactor locations were slightly different on each participant with respect to their individual body size and curvature. With equidistantly located tactors, the polar angle of each tactor may have slightly varied between individuals, an effect also previously observed (van Erp, 2005).
- Synchronicity between V and H stimuli was possibly not ecological. In the real world, visual cues outside the body, but still within the PPS, are likely seen before they come into contact with the body surface. A V stimulus being presented with a slight asynchrony, *i.e*., presented before the H stimulus, could have increased the multisensory integration effect.

### 4.3 Conclusions

The present study provided characteristics of visual and haptic sensory perceptive fields and investigated the effects of multisensory integration by combining the two. In order to develop a single unified bimodal percept, inherent differences in the encoding mechanisms of visual and haptic sensory sources must be considered. With this knowledge, appropriate transformations of each source may more effectively bridge the gap between the two corresponding reference frames. The most important contributions of this study were in demonstrating the conditions and characteristics for when bimodal VH localization performance exceeds that of the most precise or accurate unimodal condition.

Based on this work, the following conclusions can be reached:

1. Visual localization follows a radial pattern characterized by a systematic undershoot of responses that is more pronounced in the upper hemifield. Specifically, visual precision significantly decreases with eccentricity in all directions while visual accuracy asymmetrically decreases with eccentricity, particularly in the vertical direction.
2. Haptic localization follows a nonlinear pattern characterized by relative egocenters that divide the torso into separate reference frames. Haptic precision was statistically equivalent within a boundary of roughly ±8° (6 cm) of the midline, and significantly better within this region than anywhere else on the torso. Haptic accuracy was statistically equivalent on the SMP, and midlines down the center of the left and right hemifield. Together, these results affirm the processing differences on each side of the front torso region, within which sensory cues are perceived independently.
3. Performance with combined visual-haptic cues significantly improved accuracy relative to vision alone, the most reliable source, for values most extreme in the periphery (25° in absolute eccentricity in azimuth). This result confirms the hypothesis that the effect of multisensory integration is enhanced when the primary sense is degraded or impaired, *i.e*., past the bounds of ambient vision.
4. MLE predictions were statistically equivalent for bimodal precision, but incorrectly predicted bias from the least reliable source. The opposite effect was observed, as the addition of another sensory source, albeit a less reliable one, still increased the performance relative to the most reliable source alone, at least locally.

Many of the findings presented in this research align with established sensory integration theories, particularly the dominance of visual cues in most regions of peri-personal space. However, it is important to note that, to our best knowledge, no prior studies have comprehensively explored the integration of haptic and visual cues specifically in the context of localization within peri-personal space. This study, therefore, represents an important first step in filling this gap, particularly by providing empirical data that characterizes haptic and combined visual-haptic performance in both central and peripheral regions.

While the observed dominance of visual cues is consistent with established theories, our findings offer valuable insights into scenarios where haptic cues may play a complementary or even primary role. Notably, our results highlight the potential for haptics to enhance spatial perception in peripheral regions where visual efficacy decreases, or in degraded visual environments where vision is no longer the dominant sensory input. These findings point to future research directions, such as investigating the use of haptic cues in scenarios where peripheral vision or degraded visual conditions are critical, as well as optimizing the design and application of haptic feedback mechanisms to compensate for visual limitations.

In aerospace applications, these findings have direct relevance to several real-world scenarios. For instance:

- In rotorcraft operations, haptic cueing could provide critical spatial feedback during brownout or whiteout conditions, where visual cues are severely compromised due to environmental factors like sand, dust, or snow. This is currently being investigated by the PIas part of other funded research projects.
- In air-to-air combat, haptic feedback could cue the relative motion or position of an adversary aircraft, particularly when visual tracking is challenged by peripheral angles or rapid maneuvers.
- During formation flying, haptics could assist in maintaining relative positioning among aircraft, especially in low-visibility conditions or when pilots are focused on other tasks.
- In autonomous or semi-autonomous systems, haptic feedback could serve as an intuitive mechanism for conveying situational awareness, helping pilots or operators monitor and respond to spatial cues without overloading their visual channel.

By demonstrating the unique characteristics of haptic localization and its integration with visual cues, this study lays the foundation for future investigations into how haptics can be leveraged in these and other applied contexts.

## Data Availability

The raw data supporting the conclusions of this article will be made available by the authors, without undue reservation.

## Footnotes

^1^In practice, this procedure tested three unimodal conditions: visual, auditory, haptic, and all combinations of the three. Auditory data was discarded from this analysis due to insignificant results stemming from equipment flaws. The procedure, as written, disregards the auditory modality in order to maintain consistency with the following analysis and results.
