# Supplementary material for: Characterization of 2D precision and accuracy for combined visual-haptic localization
Source: Front Neurosci. 2025 Mar 12;19:1528601. doi: 10.3389/fnins.2025.1528601 (PMC11936952; doi:10.3389/fnins.2025.1528601)
Supplement: Supplementary file 1 [file Supplementary_file_1.pdf]

Characterization of 2D Precision and Accuracy for Combined Visual-Haptic Localization

Madeline Fischer [1,*], **Umberto Saetti** [2], **Martine Godfroy-Cooper** [3], **and Douglas Fischer** [4]

[1] *Department of Mathematics, University of Maryland, College Park, MD, USA*
[2] *Department of Aerospace Engineering, University of Maryland, College Park, MD, USA*
[3] *AIRBUS, Toulouse, France*
[4] *The Boeing Company, Ridley Park, PA, USA*

Correspondence*:
Umberto Saetti
saetti@umd.edu

APPENDIX

0.1 Visual Statistics

		All Eccentricities	Strictly HMP/ SMP		All Eccentricities	Strictly HMP/SMP
	Groups	p (Scheffe)	p (Scheffe)	Groups	p (Scheffe)	p (Scheffe)
Precision	R-L	0.902	0.891	U-D	0.980	0.890
	SMP-L	0.005	0.263	HMP-D	0.602	0.429
	SMP-R	0.011	0.370	U-HMP	0.699	0.324
	Regression:	$F_{(2,32)} = 6.66$ $p = 0.004$	$F_{(2,4)} = 1.93$ $p = 0.26$	Regression:	$F_{(2,32)} = 0.54$ $p = 0.59$	$F_{(2,2)} = 2.15$ $p = 0.38$
Accuracy	R-L	0.617	0.783	U-D	< 0.001	0.313
	SMP-L	0.995	0.157	HMP-D	0.973	0.719
	SMP-R	0.837	0.097	U-HMP	< 0.001	0.229
	Regression:	$F_{(2,32)} = 0.53$ $p = 0.60$	$F_{(2,4)} = 4.50$ $p = 0.09$	Regression:	$F_{(2,32)} = 15.11$ $p < 0.001$	$F_{(2,2)} = 4.01$ $p = 0.20$

Table 1. Results of VISUAL hemifield comparisons from one-way ANOVAs (with Scheffe correction), and simple linear regressions.

Not subject to U.S. Export Administration Regulations (EAR) (15 C.F.R. Parts 730-774) or U.S. International Traffic in Arms Regulations (ITAR), (22 C.F.R. Parts 120-130).

	Test	F-statistic	p-value	R^2
Precision	$\sigma^2_{xy} \sim T$	$F_{(11,23)} = 7.58$	< 0.001	0.78
	$\sigma^2_{xy} \sim \theta_T$	$F_{(27,7)} = 1.29$	0.39	0.83
Accuracy	$r \sim T$	$F_{(11,23)} = 1.15$	0.37	0.35
	$r \sim \theta_t$	$F_{(11,23)} = 1.79$	0.22	0.87

Table 2. Results of one-way ANOVAs investigating effects of polar eccentricity,T, and target direction, θ_T on VISUAL 2D precision, σ^2_{xy} and accuracy, r.

Measure	Factors	F-statistic	p-value
Precision	absTX	$F_{(3,23)} = 24.32$	< 0.001
	absTY	$F_{(2,23)} = 4.31$	0.026
	absTX*absTY	$F_{(6,23)} = 0.31$	0.928
Accuracy	TX	$F_{(3,23)} = 11.67$	< 0.001
	TY	$F_{(2,23)} = 49.86$	< 0.001
	TX*TY	$F_{(6,23)} = 6.28$	< 0.001

Table 3. Repeated measures ANOVAs investigating effects of eccentricity (with eccentricity azimuth and elevation as fixed factors) on VISUAL 2D precision and accuracy.

0.2 Haptic Statistics

		All Eccentricities	Strictly HMP/ SMP		All Eccentricities	Strictly HMP/SMP
	Groups	p (Scheffe)	p (Scheffe)	Groups	p (Scheffe)	p (Scheffe)
Precision	R-L	0.305	0.411	U-D	0.189	0.477
	SMP-L	0.005	0.201	HMP-D	0.640	0.807
	SMP-R	0.061	0.560	U-HMP	0.847	0.882
	Regression:	$F_{(2,32)} = 6.46$ $p = 0.004$	$F_{(2,4)} = 2.75$ $p = 0.18$	Regression:	$F_{(2,32)} = 1.78$ $p = 0.19$	$F_{(2,2)} = 1.10$ $p = 0.48$
Accuracy	R-L	0.281	0.242	U-D	< 0.001	0.003
	SMP-L	0.223	0.534	HMP-D	0.208	0.018
	SMP-R	0.023	0.132	U-HMP	0.272	0.020
	Regression:	$F_{(2,32)} = 4.48$ $p = 0.02$	$F_{(2,4)} = 4.20$ $p = 0.10$	Regression:	$F_{(2,32)} = 9.00$ $p < 0.001$	$F_{(2,2)} = 30.79$ $p = 0.003$

Table 4. Results of HAPTIC hemifield comparisons from one-way ANOVAs (with Scheffe correction), and simple linear regressions.

	Test	F-statistic	p-value	R^2
Precision	$\sigma^2_{xy} \sim T$	$F_{(11,23)} = 6.42$	< 0.001	0.75
	$\sigma^2_{xy} \sim \theta_T$	$F_{(27,7)} = 2.64$	0.09	0.91
Accuracy	$r \sim T$	$F_{(11,23)} = 1.42$	0.23	0.40
	$r \sim \theta_t$	$F_{(27,7)} = 3.05$	0.07	0.92

Table 5. Results of one-way ANOVAs investigating effects of polar eccentricity, T, and target direction, θ_T on HAPTIC 2D precision, σ^2_{xy}, and accuracy, r.

Measure	Factors	F-statistic	p-value
Precision	absTX	$F_{(3,23)} = 21.88$	< 0.001
	absTY	$F_{(2,23)} = 1.90$	0.07
	absTX*absTY	$F_{(6,23)} = 0.19$	0.97
Accuracy	TX	$F_{(3,23)} = 10.31$	0.006
	TY	$F_{(2,23)} = 9.36$	0.005
	TX*TY	$F_{(6,23)} = 6.08$	0.29

Table 6. Repeated Measures ANOVAs investigating effects of eccentricity (with eccentricity azimuth and elevation as fixed factors) on HAPTIC 2D precision and accuracy.

0.3 VH Statistics

		All Eccentricities	Strictly HMP/ SMP		All Eccentricities	Strictly HMP/SMP
	Groups	p (Scheffe)	p (Scheffe)	Groups	p (Scheffe)	p (Scheffe)
Precision (VH)	R-L	0.636	0.989	U-D	0.800	0.833
	SMP-L	0.003	0.265	HMP-D	0.512	0.270
	SMP-R	0.014	0.295	U-HMP	0.825	0.199
	Regression:	$F_{(2,32)} = 7.34$ $p = 0.002$	$F_{(2,4)} = 2.05$ $p = 0.24$	Regression:	$F_{(2,32)} = 0.71$ $p = 0.50$	$F_{(2,2)} = 4.2$ $p = 0.192$
Precision (MLE)	R-L	0.896	0.88	U-D	0.999	0.86
	SMP-L	0.005	0.25	HMP-D	0.688	0.43
	SMP-R	0.011	0.36	U-HMP	0.704	0.31
	Regression:	$F_{(2,32)} = 6.74$ $p = 0.004$	$F_{(2,4)} = 1.99$ $p = 0.25$	Regression:	$F_{(2,32)} = 0.44$ $p = 0.65$	$F_{(2,2)} = 2.27$ $p = 0.31$
Accuracy (VH)	R-L	0.982	0.783	U-D	< 0.001	0.158
	SMP-L	0.774	0.206	HMP-D	0.999	0.998
	SMP-R	0.689	0.236	U-HMP	< 0.001	0.212
	Regression:	$F_{(2,32)} = 0.32$ $p = 0.69$	$F_{(2,4)} = 2.60$ $p = 0.19$	Regression:	$F_{(2,32)} = 17.49$ $p < 0.001$	$F_{(2,2)} = 0.133$ $p = 0.13$
Accuracy (MLE)	R-L	0.169	0.57	U-D	0.300	0.57
	SMP-L	0.890	0.17	HMP-D	0.028	0.47
	SMP-R	0.193	0.08	U-HMP	0.022	0.24
	Regression:	$F_{(2,32)} = 2.68$ $p = 0.084$	$F_{(2,4)} = 5.09$ $p = 0.08$	Regression:	$F_{(2,32)} = 4.36$ $p = 0.021$	$F_{(2,2)} = 3.19$ $p = 0.24$

Table 7. Results of BIMODAL VH and MLE hemifield comparisons from one-way ANOVAs (with Scheffe correction), and simple linear regressions.

0.4 Modality Comparison Statistics

	Test	F-statistic	p-value	R^2
Precision (VH)	$\sigma^2_{xy} \sim T$	$F_{(11,23)} = 12.7$	< 0.001	0.86
	$\sigma^2_{xy} \sim \theta_T$	$F_{(27,7)} = 1.28$	0.39	0.83
Precision (MLE)	$\sigma^2_{xy} \sim T$	$F_{(11,23)} = 8.12$	< 0.001	0.79
	$\sigma^2_{xy} \sim \theta_T$	$F_{(27,7)} = 1.26$	0.40	0.83
Accuracy (VH)	$r \sim T$	$F_{(11,23)} = 0.77$	0.66	0.27
	$r \sim \theta_t$	$F_{(27,7)} = 5.31$	0.01	0.95
Accuracy (MLE)	$r \sim T$	$F_{(11,23)} = 3.85$	0.003	0.65
	$r \sim \theta_t$	$F_{(27,7)} = 1.24$	0.41	0.83

Table 8. Results of one-way ANOVAs investigating effects of polar eccentricity, T, and target direction, θ_T on BIMODAL VH and MLE 2D precision, σ^2_{xy}, and accuracy, r.

Measure	Factors	F-statistic	p-value
Precision (VH)	absTX	$F_{(3,23)} = 42.78$	< 0.001
	absTY	$F_{(2,23)} = 2.79$	0.08
	absTX*absTY	$F_{(6,23)} = 0.96$	0.47
Precision (MLE)	absTX	$F_{(3,23)} = 26.56$	< 0.001
	absTY	$F_{(2,23)} = 3.80$	0.04
	absTX*absTY	$F_{(6,23)} = 0.34$	0.91
Accuracy (VH)	TX	$F_{(3,23)} = 7.55$	0.002
	TY	$F_{(2,23)} = 69.49$	< 0.001
	TX*TY	$F_{(6,23)} = 5.13$	0.002
Accuracy (MLE)	TX	$F_{(3,23)} = 4.42$	0.02
	TY	$F_{(2,23)} = 7.39$	0.002
	TX*TY	$F_{(6,23)} = 1.78$	0.14

Table 9. Repeated Measures ANOVAs investigating effects of eccentricity (with eccentricity azimuth and elevation as fixed factors) on BIMODAL VH and MLE 2D precision and accuracy.

Test	t	p	Adjusted R^2
MLEAccuracy $\sim$ Wv + Wh	MLE, Wv $= -4.312$	< 0.001	0.34
VH Accuracy $\sim$ Wv + Wh	VH, Wv$= 0.39$	0.70	-0.03
VH Precision $\sim$ VP + HP	VH, VP $= 5.84$<br>VH, HP = 1.44	< 0.001<br>0.16	0.67
VH Precision $\sim$ VP + HP + Vacc + Hacc	VH, VP = 3.70<br>VH, HP $= 2.43$<br>VH, Vacc = 1.40<br>VH, Hacc = 2.23	< 0.001<br>0.02<br>0.18<br>0.034	0.70
MLEAcc $\sim$ Wv + Wh + AccV + AccH	Wv $= 14.91$<br>accV = 30.98<br>accH = 6.975	< 0.001<br>< 0.001<br>< 0.001	0.98
VHAcc $\sim$ Wv + Wh + AccV + AccH	Wv $= 0.78$<br>accV = 11.28<br>accH = -0.31	0.46<br>0.45<br>< 0.001	0.81

Table 10. Simple and multiple linear regressions investigating the relationships between precision and accuracy, and relevant predictors.
